# HSP90, as a functional target antigen of a mAb 11C9, promotes stemness and tumor progression in hepatocellular carcinoma

**DOI:** 10.1186/s13287-023-03453-x

**Published:** 2023-09-27

**Authors:** Hui-Qi Liu, Li-Xin Sun, Long Yu, Jun Liu, Li-Chao Sun, Zhi-Hua Yang, Xiong Shu, Yu-Liang Ran

**Affiliations:** 1 State Key Laboratory of Molecular Oncology, National Cancer Center/National Clinical Research Center for Cancer/Cancer Hospital, Chinese Academy of Medical Sciences and Peking Union Medical College, No. 17 Panjiayuan Subdistrict, Chaoyang, Beijing, 100021 People’s Republic of China; 2 National Center for Orthopaedics, Beijing Research Institute of Traumatology and Orthopaedics, Beijing Jishuitan Hospital, Capital Medical University, No. 31 Xinjiekou E Road, Xicheng, Beijing, 100035 People’s Republic of China

**Keywords:** Hepatocellular carcinoma, Liver cancer, Cancer stem cells, Monoclonal antibody, Tumor-associated antigens, Stemness

## Abstract

**Background:**

Identification of promising targeted antigens that exhibited cancer-specific expression is a crucial step in the development of novel antibody-targeted therapies. We here aimed to investigate the anti-tumor activity of a novel monoclonal antibody (mAb) 11C9 and identify the antibody tractable target in the hepatocellular cancer stem cells (HCSCs).

**Methods:**

The identification of the targeted antigen was conducted using SDS-PAGE, western blot, mass spectrometry, and co-immunoprecipitation. Silence of HSP90 was induced by siRNA interference. Positive cells were sorted by fluorescence-activated cell sorting. Double-immunofluorescent (IF) staining and two-color flow cytometry detected the co-expression. Self-renewal, invasion, and drug resistance were assessed by sphere formation, matrigel-coated Transwell assay, and CCK-8 assay, respectively. Tumorigenicity was evaluated in mouse xenograft models. RNA-seq and bioinformatics analysis were performed to explore the mechanism of mAb 11C9 and potential targets.

**Results:**

MAb 11C9 inhibited invasion and self-renewal abilities of HCC cell lines and reversed the cisplatin resistance. HSP90 (~ 95 kDa) was identified as a targeted antigen of mAb 11C9. Tissue microarrays and online databases revealed that HSP90 was overexpressed in HCC and associated with a poor prognosis. FACS and double-IF staining showed the co-expression of HSP90 and CSCs markers (CD90 and ESA). In vitro and in vivo demonstrated the tumorigenic potentials of HSP90. The inhibition of HSP90 by siRNA interference or 17-AAG inhibitor both decreased the number of invasion, sphere cells, and CD90^+^ or ESA^+^ cells, as well as reversed the resistance. Bioinformatics analysis and western blot verified that HSP90 activated Wnt/β-catenin signaling.

**Conclusions:**

The study preliminarily revealed the anti-tumor activity of mAb 11C9. More importantly, we identified HSP90 as a targeted antigen of mAb 11C9, which functions as an oncogene in phenotype shaping, stemness maintenance, and therapeutic resistance by activating Wnt/β-catenin signaling.

**Supplementary Information:**

The online version contains supplementary material available at 10.1186/s13287-023-03453-x.

## Background

Hepatocellular carcinoma (HCC) is a major common malignancy and a leading cause of cancer-related mortality worldwide, especially in several regions of Africa and Asia with the highest incidence rates [1, 2]. Moreover, incidence rates of HCC show geographic variations worldwide and are heavily affected by regional differences in risk factors for liver disease [3]. Unfortunately, the prognosis of patients with HCC is unsatisfactory in all regions of the world [4]. HCC is a heterogeneous tumor associated with multiple signaling pathway alterations, and the complex pathophysiology made its treatment decision challenging [5]; accordingly, despite the treatment of HCC has considerably evolved in the past decade year, survival of patients has not improved greatly due to the limited therapeutic regimens available [6]. Notably, knowledge of HCC molecular mechanisms has led to the development of systemic treatment options, such as monoclonal antibodies (mAbs), tyrosine kinase inhibitors (TKIs), and immunotherapy [7]. Therefore, much hope has been placed in the identification of novel mAbs and targets through molecular profiling.

In recent years, targeted therapy with mAbs or small-molecule kinase inhibitors has shown considerable promise in the treatment of various cancers [8]. Nevertheless, the majority of targeted therapies for HCC were ineffective and did not confer any increased therapeutic benefit, especially for those who present with advanced disease at the time of diagnosis [9]. It is exciting that cancer stem cells (CSCs) are emerging as the most promising targets for cancer treatment due to their high capabilities for self-renewal, tumorigenesis, multidrug resistance, and recurrence [10]. Thus, we proposed that the discovery and validation of novel mAbs to specifically target CSCs should be the focus of future research on HCC-targeted therapy.

Furthermore, the identification of candidate tumor-associated antigens (TAAs) exhibiting high differential cancer-specific expression is a crucial step in the development of new targeted antibody therapeutics, because the characteristics of mAbs were affected by the antigen specificity [11]. Previous research has revealed that phenotypic mAb screening followed by mass spectrometry (MS) identification of immunoprecipitated putative target antigens is an effective method to identify candidate protein targets functionally involved in cancer tumorigenesis [12, 13]. In our laboratory, a multipotent CSC mAbs library was constructed and functional screens have identified antibodies that can regulate the stemness and resistance of hepatocellular CSC. Herein, a novel function mAb designated 11C9 was selected based on its ability in regulating hepatocellular CSC stemness and resistance. In the present study, we explored the candidate target antigen of mAb 11C9 and investigated the functional properties, hoping to find exploitable therapeutic targets directly involved in the CSCs stemness phenotype for HCC-targeted therapy.

## Methods

### Cell lines and antibodies

Two human HCC cell lines (MHCC97L and BEL7402) were obtained from the Cell and Molecular Biology Laboratory, Cancer Institute, Chinese Academy of Medical Sciences (Beijing, China). Each cell line was not cross-contaminated, as identified by polymorphic short tandem repeat (STR) authentication (Additional file 1). The mAb 11C9 was obtained from the multipotent CSC mAbs library constructed in our laboratory [14].

MHCC97L cells were maintained in DMEM/H medium supplemented with 10% fetal bovine serum (FBS), L-glutamine (2 mmol/L), penicillin (100 U/mL), and streptomycin (100 μg/mL) in a humidified incubator containing 5% CO_2_ at 37 °C, whereas BEL7402 was in RIPM 1640 medium supplemented with 10% FBS, L-glutamine (2 mmol/L), penicillin (100 U/mL), and streptomycin (100 μg/mL) in a humidified incubator containing 5% CO_2_ at 37 °C. Cells were sub-cultured using 0.25% trypsin and 0.1% EDTA when confluent (> 80% confluence).

### Hepatocellular CSCs (HCSCs) culture

HCSCs were, respectively, enriched from MHCC97L and BEL7402 cells in the serum-free sphere induction medium [15]. In brief, MHCC97L and BEL7402 cells were sub-cultivated for 48 h and centrifuged to collect cell sediment. Subsequently, cells were re-suspended in a serum-free DMEM/F12 medium containing epidermal growth factor (EGF; 20 ng/ml), basic fibroblast growth factor (bFGF; 20 ng/ml), leukemia inhibitory factor (LIF; 10 ng/mL), B27 (1:50), L-glutamine (2 mmol/ml), and methylcellulose (0.8%) for sphere induction. Cells were seeded in an ultra-low attachment flask at a density of 2 × 10^4^ cells/ml and maintained at 37 °C in a humidified atmosphere of 5% CO_2_ in the air. From day 2, induction was begun through supplementing medium every 2 days. After the sphere formation (day 7–10), sphere cells were harvested and dissociated into single cells with trypsin for the subsequent experiments.

### LC‐MS/MS identification

The mAb11C9-targeted antigen was purified using agarose G (GE, Boston, USA) and long-range SDS-PAGE, as described previously [16]. Briefly, SDS-PAGE was visualized using the Coomassie brilliant blue staining and Western blot, respectively. The detected band was excised and then measured by the liquid chromatography-MALDI-tandem time of flight (LC-MALDI-TOF/TOF). Mascot database (http://www.matrixscience.com) was used for fragment sequences analysis. Finally, the identified band was further confirmed by co-immunoprecipitation (Co-IP) using the purified mAb 11C9, a commercial antibody to HSP90 (4877S, CST), or their corresponding negative control (normal mouse IgG and rabbit IgG).

### Tissue microarray and immunohistochemistry (IHC)

The IHC microarray BC03119 (including 103 HCC and 12 normal samples) and LV805 (including 10 hepatitis and 40 liver cirrhosis samples) were purchased from Alenabio Biotechnology (Shanxi, China). HCC survival microarray HLiv-HCC180Sur-04 (including 76 HCC samples) was purchased from Shanghai Outdo Biotechnology (Shanghai, China). A standard IHC staining protocol was followed to detect the HSP90 expression using a commercial UItraSensitiveTM S-P Kit (Maxim Biotechnologies, China). HSP90 expression was evaluated using a semi-quantitative scoring system established based on the staining intensity (1 = none, 2 = weak, 3 = moderated, 4 = intense) and percentage of positively stained cells (< 5% vs. ≥ 5%). IHC staining of samples was stratified as either positive HSP90 expressing (≥ 5% and moderated/intense staining) or negative HSP90 expressing (< 5% and none/weak staining).

### Flow cytometry and fluorescence-activated cell sorting (FACS)

The expression of cell surface proteins CD90, EAS, and HSP90 and the cell sorting were conducted by flow cytometry. Briefly, parental or sphere cells (100 × 10^6^ density) were dissociated with 0.25% trypsin and re-suspended with a medium containing FBS. Then, cells were incubated with fluorescein isothiocyanate (FITC)-conjugated CD90 (eBioscience), phycoerythrin (PE)-conjugated ESA (eBioscience), and PE_Cy5.5-conjugated HSP90 or FITC-conjugated HSP90 alone, or in combination (as indicated in the figure) at room temperature for 1 h. The standard was established by isotype match control. The percentage of expression was calculated by comparing to the respective isotype control. The labeled cells were analyzed using FACSAria (BD Biosciences). HSP90^+^, HSP90^−^, CD90^+^, CD90^+^HSP90^+^, CD90^−^HSP90^+^, CD90^+^HSP90^−^, CD90^−^HSP90^−^, ESA^+^, ESA^+^HSP90^+^, ESA^−^HSP90^+^, ESA^+^HSP90^−^, and ESA^−^HSP90^−^ cells were sorted using a FACSAria cell sorter (BD Biosciences). To determine the co-expression of CSCs makers and HSP90 in CSCs, we calculated the rate of double-positive cells among single-positive cells. Double-positive rate (%) was defined as the proportion of CD90/HSP90 or ESA/HSP90 double-positive cells (Q2) in all single-positive cells of CD90 or ESA (Q1 + Q2), thus calculated as Q2/(Q1 + Q2) × 100%.

### Cells treatment and siRNA interference-mediated knockdown of HSP90

MHCC97L and BEL7402 cells were exposed to various concentrations (0.2, 0.4, 0.8, and 1.0 mg/ml) of mAb 11C9 to detect its anti-tumor activity. The HSP90 expression in sphere cells was inhibited by siRNA interference or small-molecule inhibitor 17-AAG (20, 80, and 320 nM) to explore the role of HSP90 involving stemness features of HCSCs.

The siRNA against HSP90 (siRNA-1: 5′-UUUAGUACCAGACUUGGCAAUGGUU-3′ or siRNA-2: 5′-AACCAUUGCCAAGUCUGGUACUAAA-3′), and its stable non-specific siRNA (negative control) were provided by Invitrogen (Beijing, China). The siRNA transfection was performed with Lipofectamine™ 2000 Transfection Kit (Thermofisher, USA). After transfection, the knockdown of HSP90 was confirmed by WB.

### Sphere formation, cell invasion, and cell viability assay

The above FACS-sorted, transfected, or mAb 119C/17-AAG-treated sphere cells were collected for subsequent experiments measuring self-renewal and cell invasion ability, and cell viability. The self-renewal ability was evaluated using sphere formation assay in serum-free DMEM/F12 medium. Sphere cells were counted using an inverted microscope (Nikon, Japan). Cell invasion was evaluated using the matrigel-coated Transwell chamber (8 μm, Corning, USA). Invaded cells were fixed with methanol and acetone, followed by staining with DAPI. Images were captured under a fluorescent microscope (Nikon, Japan). CCK-8 assay was performed to determine the cell sensitivity to cisplatin (0.125–8 μg/ml). The optical density (OD) was detected at 450 nm under a microplate reader (Model 450, Bio-Rad, USA). The half maximal inhibitory concentration (IC_50_) of cisplatin was determined.

### Double-immunofluorescent (IF) staining

Double-IF staining was performed, respectively, in live cells and fixed cells to identify co-localization of HSP90 with cell surface markers for CSCs, CD90 in MHCC97L cell lines [17] or ESA in BEL7402 cell lines [18], according to the standard protocol. Primary antibodies targeting HSP90 (purified mAb 11C9 or commercial antibody) as well as secondary antibodies prelabeled with Cyanine Cy™3 Goat Anti-Mouse IgG + IgM (H + L) (red) or Alexa Fluor®488 Goat Anti-Rabbit IgG (H + L) (green) were used in this study. For the direct double-IF staining of HSP90 and CD90/ESA, fluorescent-labeled antibodies including FITC-conjugated CD90 (green), PE_Cy5.5-conjugated HSP90 (red), PE-conjugated ESA (red), and FITC-conjugated HSP90 (green) were used. DAPI (blue) was used for nuclear staining. Fluorescent images were obtained under fluorescence microscopy (Nikon, Japan) or a confocal laser scanning confocal microscope (Leica Microsystems, Germany). As PKH26–positive cells exhibit greater in vivo tumor-initiating capacity and stem cell frequency [19], PKH26 was co-stained with HSP90 to investigate the role of HSP90 in CSCs (Additional file 2: Supplementary Method).

### Western blot

Western blot was performed according to the standard protocol using the primary antibodies against HSP90 (4877S, CST), β-catenin (9562S, CST), phosphorylated β-catenin (pβ-catenin, #9566, CST), phosphorylated GSK-3α/β (9331S, CST), and GAPDH (97166, CST; endogenous control), as well as the corresponding HRP-conjugated secondary antibodies, HRP-conjugated goat anti-mouse IgG (Jackson, USA) and HRP-conjugated goat anti-rabbit IgG (Jackson, USA). Protein bands were developed with an enhanced chemiluminescence detection kit (Millipore, USA). Finally, the band intensity of target proteins was semi-quantified after normalization with the intensity of endogenous control using Bandscan software.

### Nude mice tumorigenicity assay

Specific-pathogen-free BALB/C nude mice (female, 4 weeks old) were purchased from Charles River Laboratories (Beijing, China). All animals were housed at the Cancer Hospital, Chinese Academy of Medical Sciences (Beijing, China) and in standard conditions (60 ± 5% humidity, 25 ± 1 °C temperature, and 12-h light/dark cycle). All animal procedures were conducted in accordance with the NIH Guide for the Care and Use of Laboratory Animals and authorized by the institutional review board (Ethical Approval: NCC2017A014) before the start of any protocol-specified procedures. BALB/C nude mice were randomly allocated into 9 groups (7 in each group). FACS-sorted cells were implanted into the forelimb axilla of mice in different doses (2 × 10^2^, 2 × 10^3^, and 2 × 10^4^) under sterile conditions. Tumor growth was monitored every 14 days until 10 weeks after the inoculation. Finally, mice were euthanized with cervical dislocation under anesthesia with isoflurane (1.5–2%).

### Bioinformatics analysis

RNA-sequencing data of HCC were downloaded from the TCGA database. Patients were divided into HSP90-high and HSP90-low groups according to the median value of HSP90 expression. The IHC staining of HSP90 in normal and HCC patients was obtained from The Human Protein Atlas (THPA) database (https://www.proteinatlas.org/).

To investigate the mechanism of mAb 11C9’s anti-tumor activity and potential targets of HSP90, differentially expressed genes (DEGs) between CD90^+^HSP90^+^ and CD90^+^HSP90^−^ cells, as well as CD90^+^HSP90^+^ and 11C9-treated CD90^+^HSP90^+^ cells, were identified. The mRNA was extracted from each sample and subjected to transcriptome analysis using Affymetrix GeneChip 3'IVT expression array by Capitalbio Corporation (Beijing, China). Sequencing results were available from GEO database (GSE215984). The AGCC software was used to save the chip fluorescence scanning image into a. DAT file for analysis. Statistical software R was used to statistical calculation and interpretation of DEGs. DEGs were defined as those with q-value < 0.05 and Log_2_ fold change ≥ 2 or ≤ 0.5. Biological significance of DEGs was explored by GO terms enrichment analysis, including biological process (BP), cellular component (CC), and molecular function (MF), based on DAVID (https://david.ncifcrf.gov/). Kyoto Encyclopedia of Genes and Genomes (KEGG) pathway enrichment analysis was also performed with DAVID (https://david.ncifcrf.gov/). Protein–protein interaction (PPI) network analysis was performed using Cytoscape software.

### Statistical analysis

SPSS statistics version 17.0 (SPSS Inc., USA) was used for statistical analysis. Experimental data were presented as the mean ± standard error of the mean (S.E.M). All in vitro experiments were independently repeated a minimum of three times; the in vivo experiments were performed on 7 mice from each group. Clinical data of patients were presented as mean ± standard deviation (s.d.) or number (percentage, %). Statistical significance between two groups was analyzed by student’s t test, and among multiple groups was analyzed by one-way or two-way analysis of variance (ANOVA) with Bonferroni post hoc test. Survival analysis was determined using Kaplan–Meier method and compared using the log-rank test. Cox proportional-hazards model was used to evaluate the independent prognostic factors after adjustment of baseline data (age, gender, stage, etc.). A *p* values of < 0.05*, < 0.01**, and < 0.001*** were considered statistically significant.

## Results

### mAb 11C9 inhibited the stemness and malignant behavior of HCSCs in vitro

**Fig. 1:**
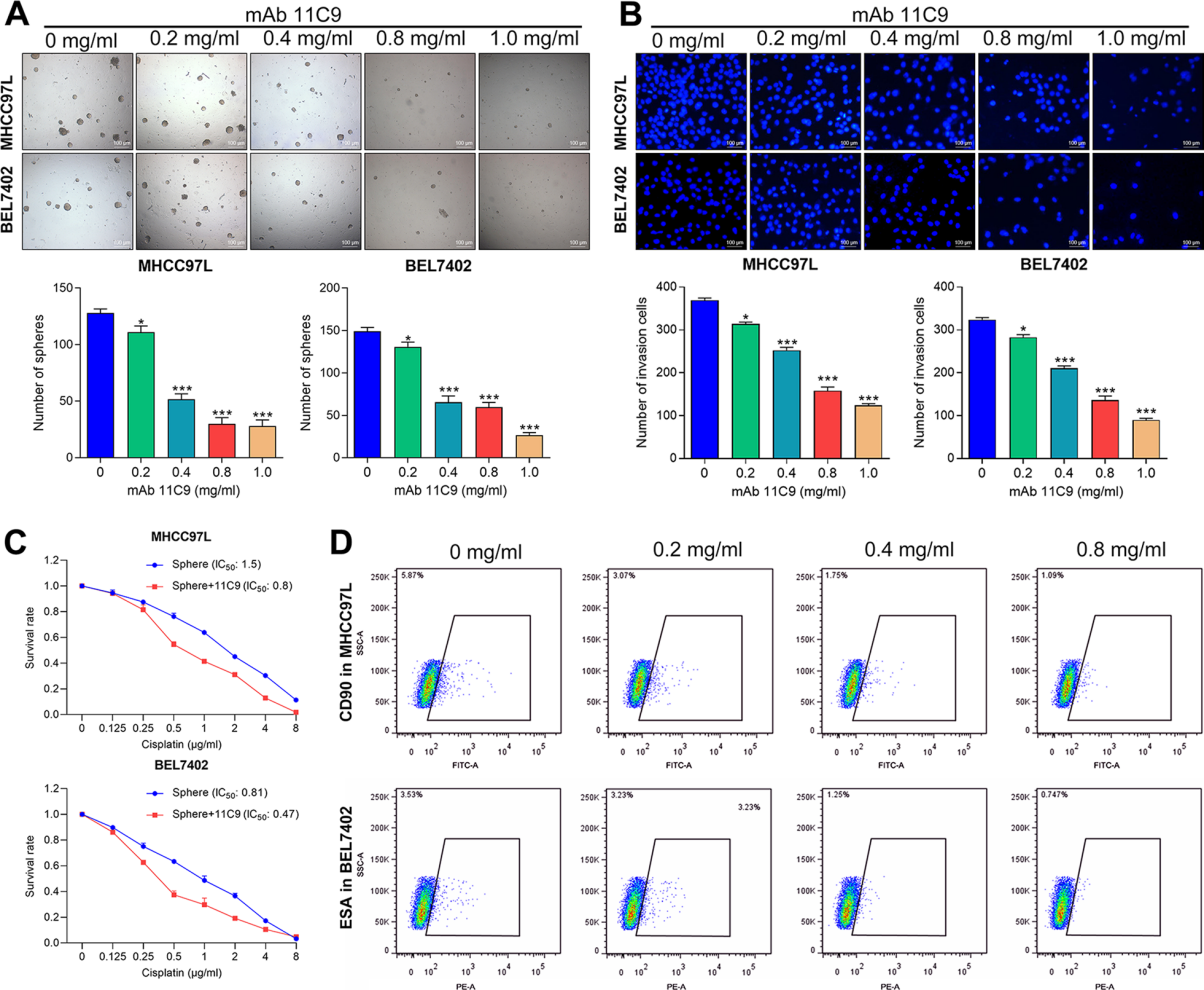
mAb 11C9 inhibited the stemness and malignant behavior of hepatocellular CSCs in vitro*.* Two human HCC cell lines (MHCC97L and BEL7402) were induced to hepatocellular CSCs and exposed to various concentrations (0–1.0 mg/ml) of mAb 11C9 for following experiments. **A** Representative images (scale bar, 100 μm) of sphere formation assay and quantitative results showed the inhibition effects of mAb 11C9 on self-renewal ability in MHCC97L and BEL7402 sphere cells. **B** Representative images (scale bar, 100 μm) of matrigel-coated Transwell assay and quantitative results showed the inhibition effects of mAb 11C9 on cell invasion ability in MHCC97L and BEL7402 sphere cells. **C** CCK-8 assay was performed and the IC_50_ value was calculated to evaluate the cell resistance. **D** Flow cytometry assay showed the inhibition effects of mAb 11C9 on cancer stem cell markers in sphere cells. Data plotted represent the mean ± S.E.M of at least three independent experiments. Statistics: *p < 0.05, ***p < 0.001

To explore the anti-tumor activity of the mAb 11C9 that was obtained from the multipotent CSC mAbs library, two human HCC cell lines (MHCC97L and BEL7402) were induced to CSCs and exposed to various concentrations of mAb 11C9. As shown in Fig. 1A, mAb 11C9 significantly inhibited the sphere formation ability both in MHCC97L and BEL7402 sphere cells, as evident by the decrease in sphere size and number. Meanwhile, the invasion ability of two sphere cells was suppressed after mAb 11C9 exposure (Fig. 1B). Given that the dose of 0.8 mg/ml mAb 11C9 was enough to induce a considerable change in cell phenotypes, the doses ranged from 0.2 mg/ml to 0.8 mg/ml mAb 11C9 were used to treat the sphere cells in cell viability assay. As a result, mAb 11C9-treated MHCC97L cells (IC_50_ = 0.8) were more sensitive to cisplatin treatment compared to the control (IC_50_ = 1.5), displaying a significantly reduced IC_50_ value (Fig. 1C). Similar result was observed in the BEL7402 cell (IC_50_ = 0.47 in mAb 11C9-treated cells vs. 0.81 in control; Fig. 1C). However, low-doses (0.2 and 0.4 mg/ml) of mAb 11C9 had no effects on the cisplatin sensitivity in HCSCs (data not shown). Furthermore, the FACS expression analysis of CSCs markers in sphere cells revealed that mAb 11C9 inhibited the expression of CD90 (known as CSCs marker for MHCC97L cells) and ESA (known as CSCs marker for BEL740 cells) in a dose-dependent manner (Fig. 1D). Thus, these results preliminarily demonstrated that the obtained mAb 11C9 has anti-tumor activity, presenting as the inhibition of the stemness and invasion abilities, as well as the improved chemotherapeutic treatment.

### Identification of HSP90 identified as a target antigen for mAb 11C9

**Fig. 2:**
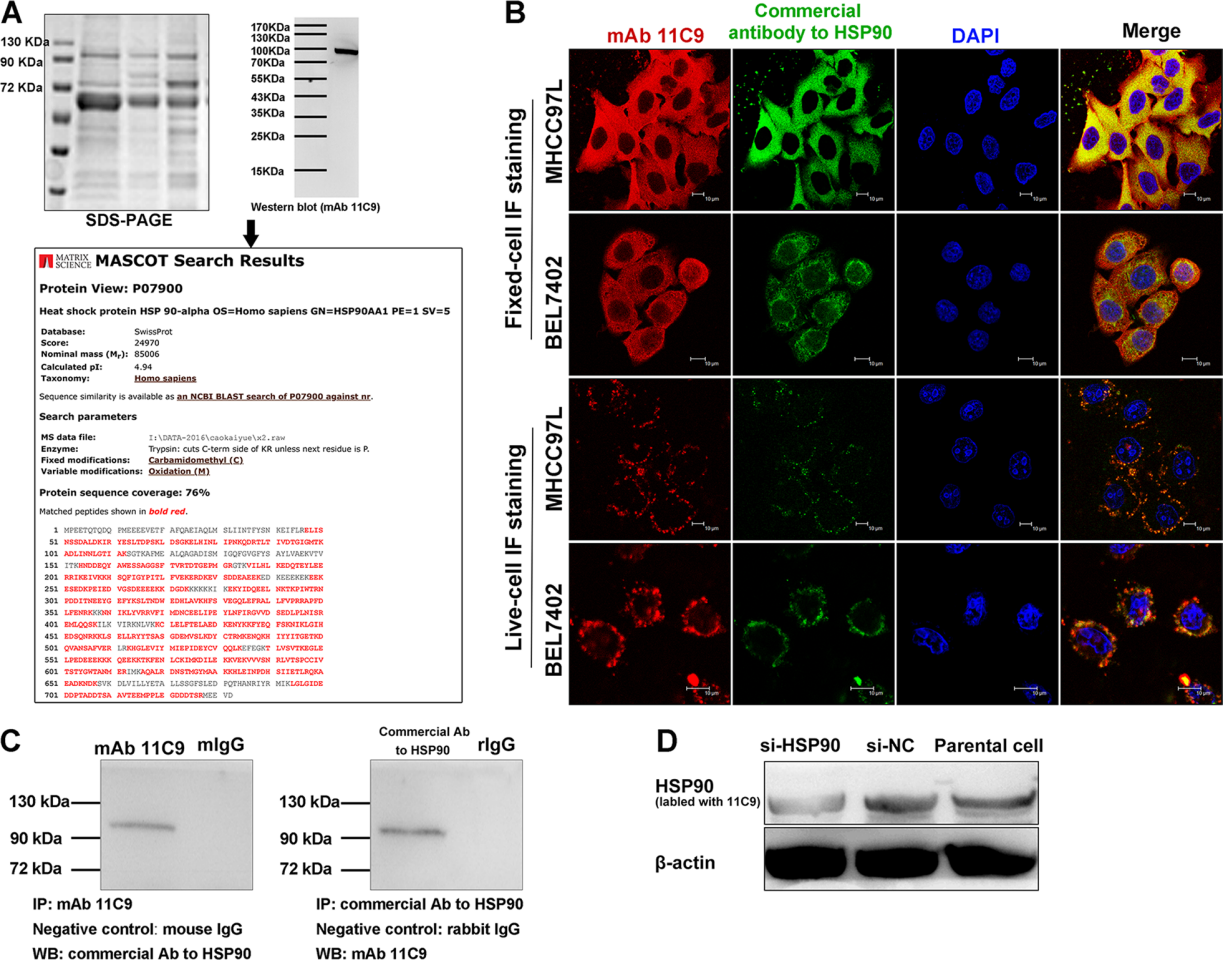
Identification of HSP90 identified as a target antigen for mAb 11C9. **A** Targeted antigen recognized by mAb 11C9 was identified by sequentially using SDS-PAGE, western blot, and mass spectrometry. The 95 kDa band was identified as human HSP90 by analysis in Mascot database. **B** Double-immunofluorescence staining showed the co-localization of antigen recognized by mAb 11C9 (red) or commercial antibody to HSP90 (green). scale bar: 10 μm. **C** Co-immunoprecipitation/Western blot confirmed that the 95 kDa band interacted with mAb 11C9. **D** After the siRNA interference of HSP90, Western blot showed a reduction of HSP90 when probed with mAb 11C9. Full-length blots/gels are presented in Additional file 2: Figure S1

To isolate and identify the candidate target antigen of mAb 11C9 in MHCC97L cells, protein identification was conducted using MS analysis, followed by double IF staining and Co-IP. Whole-cell lysates were purified by mAb 11C9-linked sepharose 4B, electrophorized by SDS-PAGE, and immunoblotted with mAb 11C9. Under the conditions we used, mAb 11C9 detected one protein with *M*_*r*_ of ~ 95 kDa in lysates, as shown in Fig. 2A (Full-length blots/gels are shown in Additional file 3: Fig S1). Through the MS analysis and searching in the Mascot database (Fig. 2A), this 95 kDa band was identified as human HSP90α (Mascot score, 24970; Estimated molecular weight/pI, 85006/4.94; Protein sequence coverage, 76%).

The subsequent double-IF staining showed that the 11C9-labeled target antigen was well-fitted to that labeled with commercial antibody to HSP90 and co-located on the cell membrane (Fig. 2B). Co-IP further confirmed that the 95 kDa band was either interacted with 11C9 or commercial antibody to HSP90 (Fig. 2C; Full-length blots/gels are shown in Additional file 3: Fig S1). An obvious reduction in the HSP90 expression was observed in the si-HSP90 transfected cells when probed with 11C9 (Fig. 2D; Full-length blots/gels are shown in Additional file 3: Fig S1). Taken together, these results identified HSP90 as the targeted antigen of mAb 11C9.

### HSP90 predicts a worse outcome for patients with HCC

**Fig. 3:**
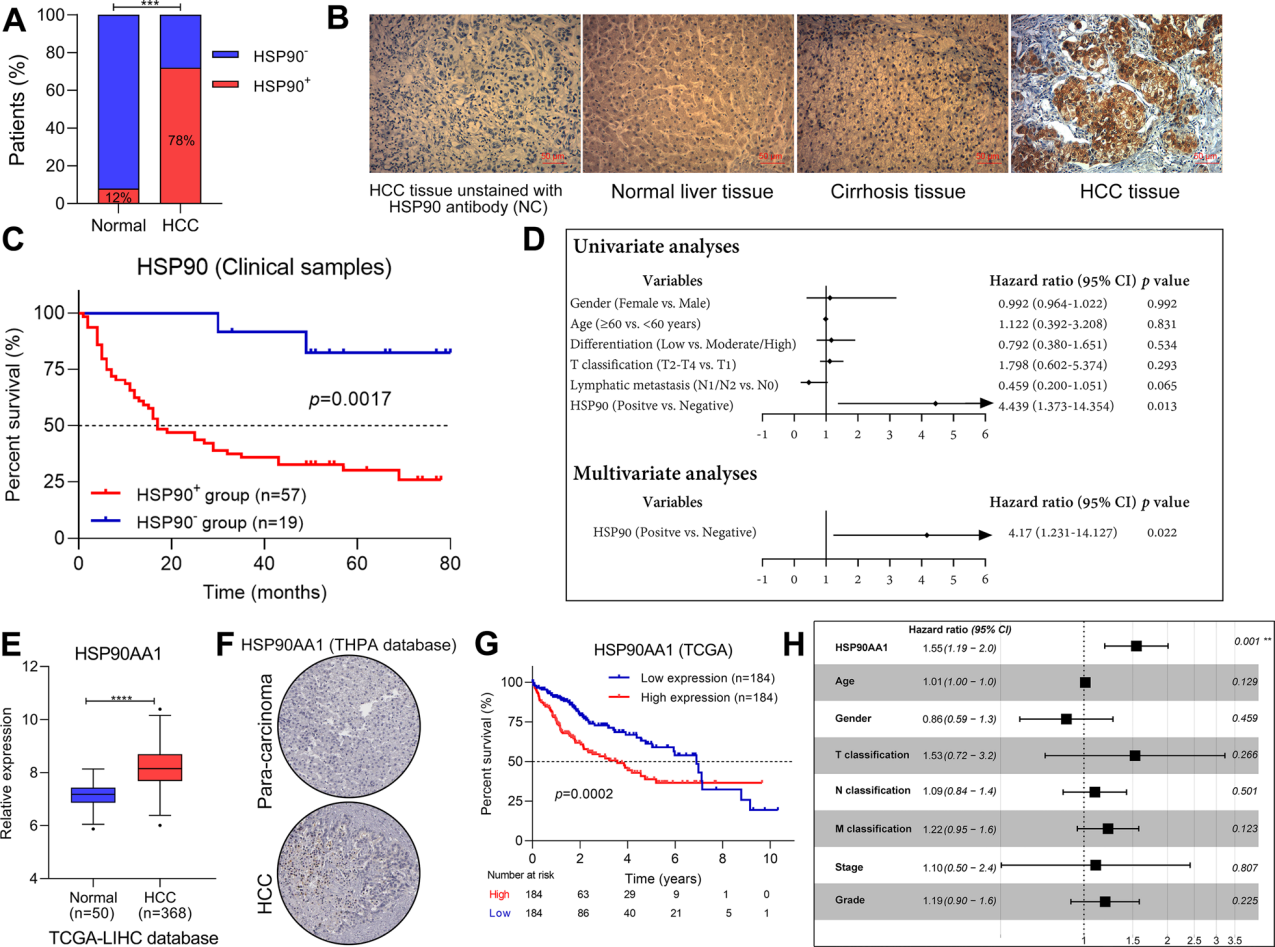
HSP90 predicts a worse outcome for patients with HCC. **A** The distribution of subjects with HSP90-positive expression in HCC and normal participants. **B** The HSP90 expression in liver tissues was detected using IHC and representative images were shown. Scale bar, 50 μm. **C** Kaplan–Meier curve of overall survival in 76 HCC patients, classified by HSP90 expression (HSP90^+^, positively stained cells ≥ 5% and moderated/intense staining; HSP90^−^, < 5% and none/weak staining). **D** Forest plot of the Cox-regression analysis on overall survival in 76 HCC patients. **E** Expression of HSP90 in HCC patients that derived from the TCGA database. **F** Expression of HSP90 in HCC patients that derived from the THPA database. **G** Kaplan–Meier curve of overall survival in the TCGA database, classified by HSP90 expression (high, FPKM values above the median; low, FPKM values below the median). **H** Forest plot of the Cox-regression analysis on overall survival as per the data derived from the TCGA database. *NC* negative control. Statistics: ***p < 0.001

To investigate the clinical significance of HSP90 in the HCC progression, we analyzed the expression of HSP90 and its correlation with survival outcomes and clinicopathological features. IHC in tissue microarrays revealed that the positive expression of HSP90 in HCC is more remarkable compared to normal tissue (Fig. 3A), and is specific in tumor tissue (Fig. 3B). According to the expression status of HSP90, tumors were stratified as positive HSP90 expressing or negative HSP90 expressing. A high association was observed between HSP90 expression and tumor differentiation or metastasis (*p* < 0.05, Additional file 2: Table 1S), as well as the poor prognosis of HCC patients (Fig. 3C). Furthermore, the Cox-regression analysis confirmed that HSP90 expression is an independent risk factor for the overall survival (Fig. 3D). Consistently, the high expression of HSP90 (Fig. 3E, F) and its association with the prognosis of HCC patients (Fig. 3G, H) were also verified in online databases (TCGA, https://www.cancer.gov/tcga; THPA, https://www.proteinatlas.org/). Overall, these results suggested that HSP90, acting as a risk factor, could independently evaluate the survival prognosis of patients with HCC.

### HSP90 is identified as a stemness-related protein in HCSCs

**Fig. 4:**
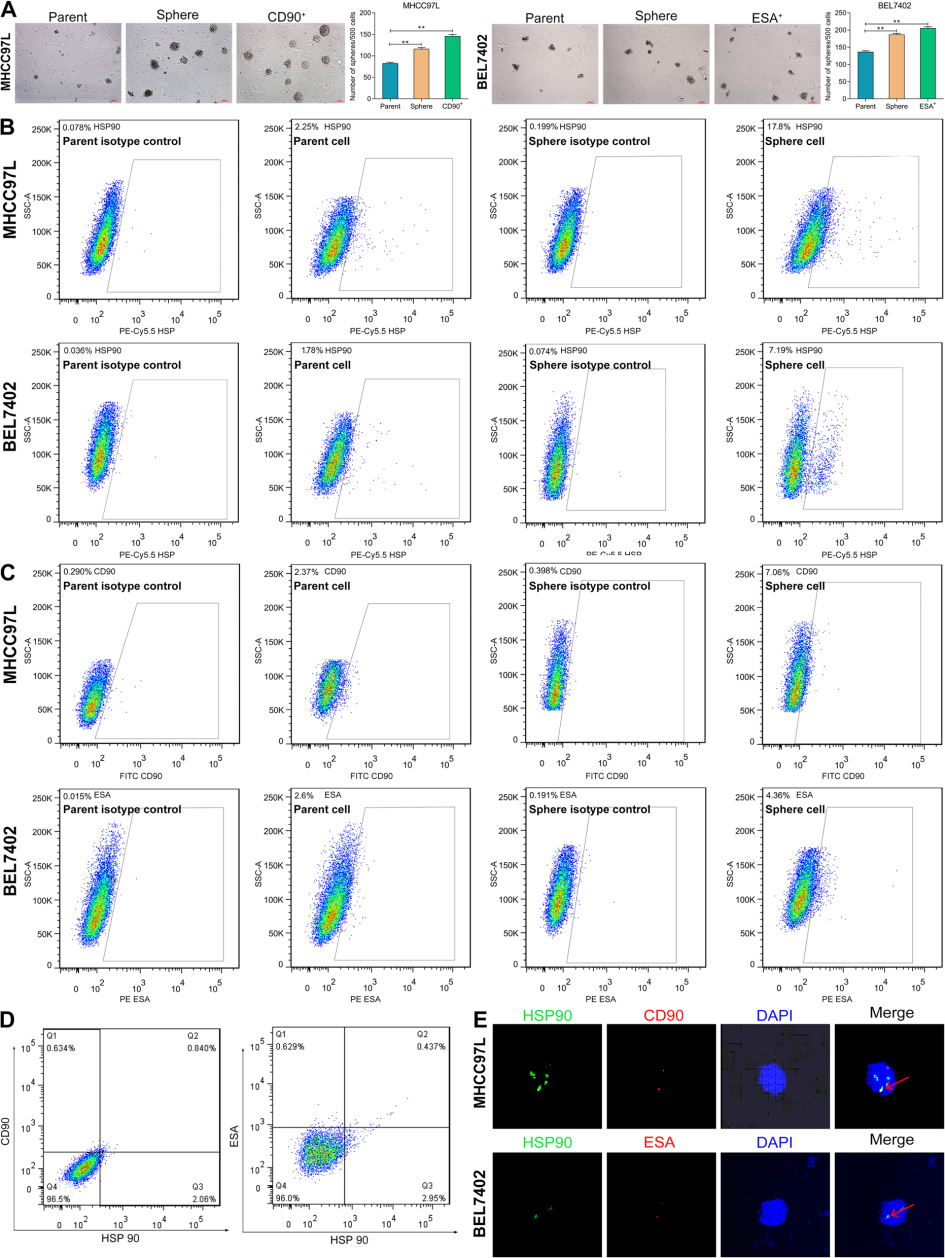
HSP90 is identified as a stemness-related protein in HCSCs. **A** Sphere formation assay revealed the enhanced self-renewal abilities of spheres and HCSCs. scale bar, 100 μm. **B**, **C** Flow cytometry assay verified the overexpression of HSP90, CD90, and ESA in spheres. **D** Two-color flow cytometry showed the co-expression of HSP90 and CD90 or ESA in MHCC97L (left panel) and BEL7402 (right panel) sphere cells, respectively. **E** Double-IF staining revealed the co-location of HSP90 and CSCs markers (CD90 or ESA). Data plotted represent the mean ± S.E.M of at least three independent experiments. Statistics: **p < 0.01

To identify the role of HSP90 in HCSCs, sphere-forming culture was performed using HCC cell lines, MHCC97L (CSC marker, CD90), and BEL7402 (CSC marker, ESA); and then CD90^+^ and ESA^+^ sphere cells were selected using the FACS sorting. As shown in Fig. 4A, the number of colonies formed by sphere cells, especially CD90^+^ and ESA^+^ sphere cells, was superior to those formed by parental cells both in MHCC97L and BEL7402 cell lines, indicating the stem cell-like features of sphere cells. In Fig. 4B, FACS analysis revealed that HSP90 was overexpressed in the membrane surface of sphere cells in the MHCC97L (17.8% vs. 2.25%) and BEL7402 cells (7.19% vs. 1.78%), compared to their corresponding parent cells. Meanwhile, CSC markers, CD90 (7.06% vs. 2.37%) and ESA (4.36% vs. 2.6%), showed a similar tendency of high expression with HSP90 in sphere cells (Fig. 4C), implying the potential co-expression of HSP90 and stem cell markers. Furthermore, two-color flow cytometry (Fig. 4D) confirmed this co-expression by determining the co-staining of CSCs makers and HSP90 in CSCs, with an HSP90/CD90 double-positive rate of 57.1% in CD90^+^MHCC97L cells, and an HSP90/ESA double-positive rate of 40.9% in ESA^+^BEL7402 cells. As indicated in Fig. 4E, double-IF staining also demonstrated that HSP90 was co-expressed with CSCs markers, CD90 or ESA. Additionally, the quiescent PKH26-positive cells with stem-like characteristics were also co-labeled with HSP90 (Additional file 2: Fig. S1). Based on the co-expression of HSP90 with CSCs markers or PKH26, we speculated that HSP90 might be a stemness-related protein in HCSCs.

### HSP90 promoted stemness features of HCSCs and tumor progression in HCC

**Fig. 5:**
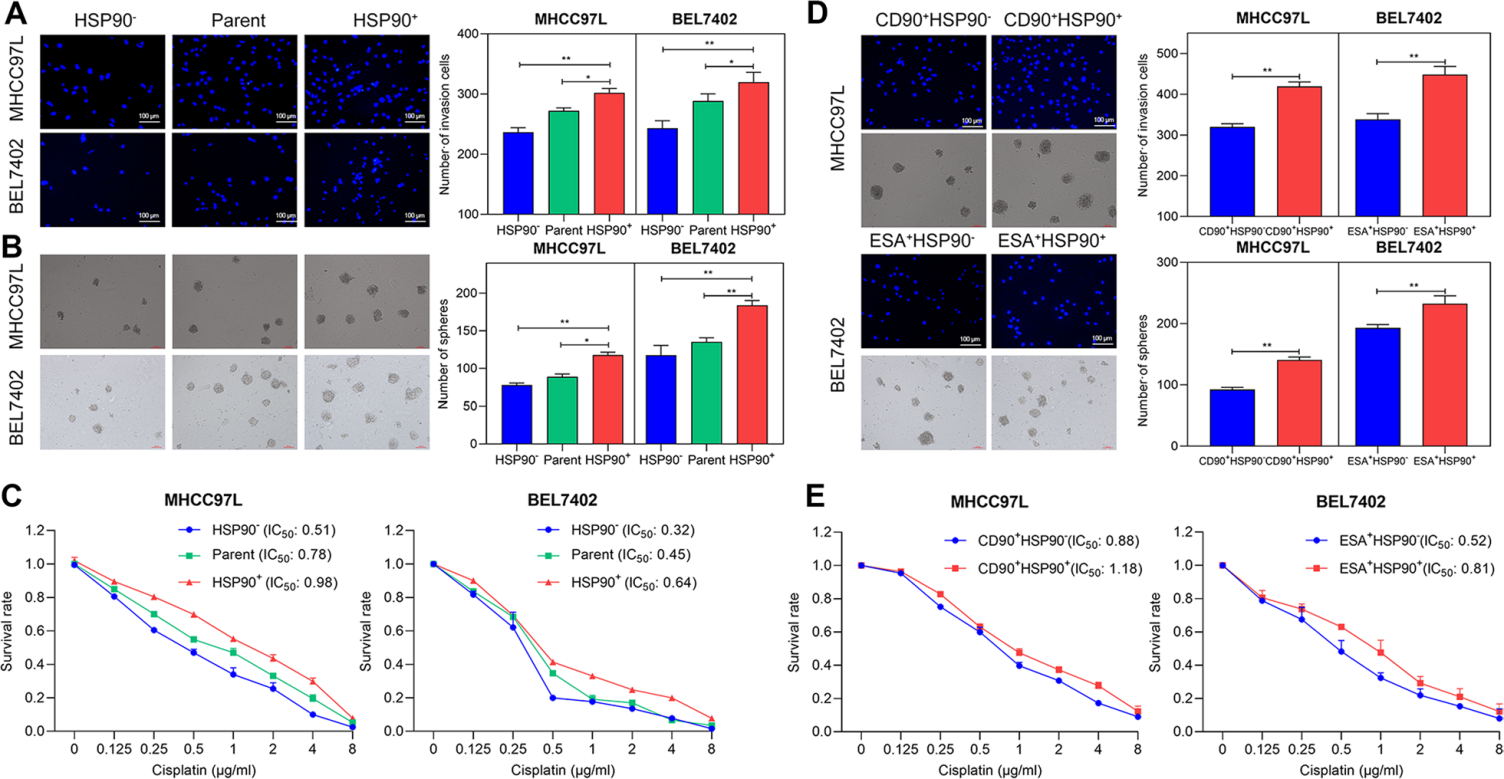
HSP90 promoted the malignant phenotypes of HCC cell lines. FACS was conducted to sort the HSP90^−^, HSP90^+^, CD90^+^HSP90^−^, CD90^+^HSP90^+^, ESA^+^HSP90^−^, ESA^+^HSP90^+^ cells, using parental cells as control. **A,** **B**, **D** Matrigel-coated Transwell assay and sphere formation assay measured the invasion and self-renewal abilities, respectively. **C**, **E** CCK-8 assay detected drug resistance. Data plotted represent the mean ± S.E.M of at least three independent experiments. Statistics: *p < 0.05, **p < 0.01

In order to assess the function of HSP90 in tumor progression and stemness features of HCSCs, we first sorted HSP90^+^, CD90^+^HSP90^+^, and ESA^+^HSP90^+^ cells and compared the self-renewal, invasion, resistance, and tumorigenicity to the parent or negative cells. As shown in Fig. 5A-5C, both HSP90^+^ MHCC97L, and HSP90^+^ BEL7402 showed significantly higher abilities of invasion (Fig. 5A), self-renewal (Fig. 5B), and higher cisplatin resistance (Fig. 5C) when compared to parent or HSP90^−^ cells. The in vivo tumorigenicity assay revealed that tumor formation could be initiated by 200 HSP90^+^ cells but 2 × 10^4^ HSP90^−^ cells both in MHCC97L and BEL7402 cell lines (Table 1), further suggesting the tumorigenic potentials of HSP90.

**Table 1:** Tumorigenicity assay of HSP90+, HSP90− and parent cells

Cell types	2 × 10^2^	2 × 10^3^	2 × 10^4^
MHCC97L			
HSP90^+^	1/7	3/7	5/7
HSP90^−^	0/7	0/7	1/7
Parent	0/7	1/7	3/7
BEL7402			
HSP90^+^	1/7	3/7	5/7
HSP90^−^	0/7	0/7	1/7
Parent	0/7	1/7	3/7

**Fig. 6:**
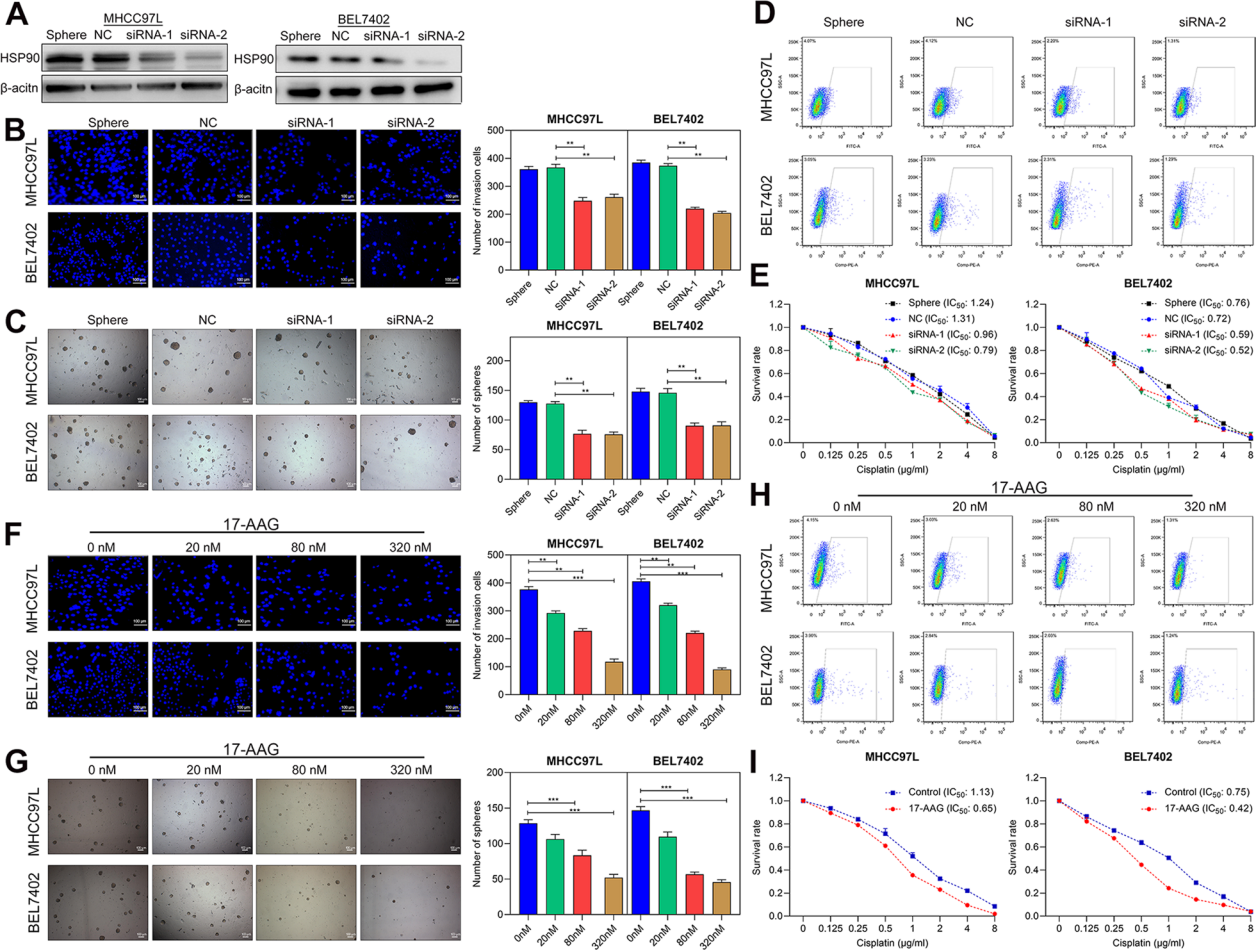
HSP90 promoted the stemness features of HCSCs. **A** Western blot verified the knockdown of HSP90 in both MHCC97L and BEL7402 sphere cells. Full-length blots/gels are presented in Additional file 2: Figure S2. **B**, **F** Matrigel-coated Transwell assay showed the reduction in invasion ability by si-HSP90 or 20–320 nM 17-AAG (inhibitor for HSP90). scale bar, 100 μm. **C**, **G** Sphere formation assay showed the reduction in sphere cells by si-HSP90 or 17-AAG. Scale bar, 100 μm. **D**, **H** Flow fluorescence assay revealed the inhibition of CD90^+^ ESA^+^ cells expression by si-HSP90 or 17-AAG. **E**, **I** CCK-8 assay of drug resistance showed the decrease of IC_50_ value by si-HSP90 or 17-AAG. Data plotted represent the mean ± S.E.M of at least three independent experiments. Statistics: *p < 0.05, **p < 0.01, ***p < 0.001

In the HCSCs sorted by CSCs markers (CD90 or ESA), we also observed the higher abilities of invasion (Fig. 5D), self-renewal (Fig. 5D), and cisplatin resistance (Fig. 5E) of CD90^+^HSP90^+^ and ESA^+^HSP90^+^ cells compared to HSP90^−^ cells, implying that HSP90 promoted stemness features of HCSCs. Therefore, to further confirm this above finding, we suppressed the HSP90 expression in the sphere cells by siRNA interference (Fig. 6A; Full-length blots/gels are shown in Additional file 3: Fig. S2) or adding small-molecule inhibitor 17-AAG (20–320 nM) for HSP90. As a result, the inhibition of HSP90 induced a decrease in invasion cells (Fig. 6B, F), the size and number of sphere cells (Fig. 6C, G), and the expression of CD90^+^ or ESA^+^ cells (Fig. 6D, H). In addition, the inhibition of HSP90 increased the sensitivity to cisplatin, with a significant decrease in IC_50_ values of sphere cells (Fig. 6E, I). Meanwhile, we observed that the 17-AAG-mediated inhibition occurred in a dose-dependent manner (Fig. 6F–H). Overall, these results suggested that HSP90 could promote the stemness features of HCSCs and then accelerate tumor progression in HCC.

### mAb 11C9 plays anti-tumor activity by inhibiting HSP90/Wnt/β-catenin signaling

**Fig. 7:**
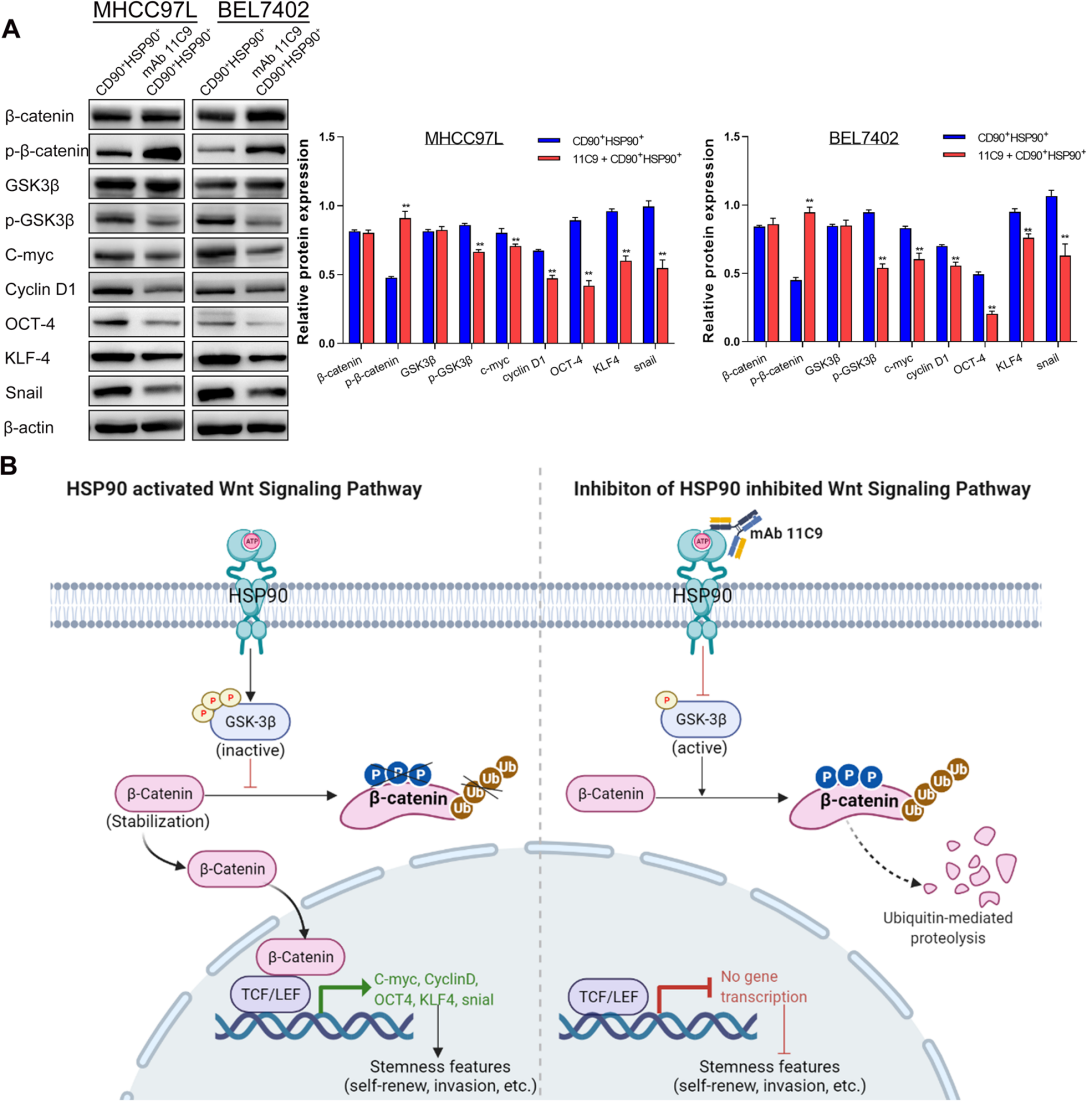
mAb 11C9 plays anti-tumor activity by inhibiting HSP90/Wnt/β-catenin signaling. **A** Western blot verified the expression of proteins in Wnt/β-catenin signaling or its downstream proteins after inhibition of HSP90 by mAb 11C9. Data plotted represent the mean ± S.E.M of at least three independent experiments. Full-length blots/gels are presented in Additional file 2: Figure S3. Statistics: *p < 0.05, **p < 0.01. **B** The potential molecular mechanism of the anti-tumor activity of mAb 11C9 in HCC

Subsequently, we performed the bioinformatics analysis to investigate the potential targets of HSP90-induced biological properties of HCSCs. By comparing DEGs between CD90^+^HSP90^+^ and CD90^+^HSP90^−^ cells, we identified 126 up-regulated DEGs with a fold change of > twofold (see Additional file 2: Fig. S2A). GO and KEGG analysis showed that DEGs were involved in the regulation of cell cycle and mitosis, such as microtubule binding, cell cycle, cell growth, microtubule cytoskeleton, and p53 signaling pathway. (Additional file 2: Fig. S2B & S2C). Furthermore, the PPI network analysis showed a strong interaction among these cell cycle/mitosis-related proteins (Additional file 2: Fig. S2D). According to these results, we speculated that HSP90 mainly participated in the self-renewal of HCSCs by regulating the cell cycle and mitosis. For this hypothesis verification, we down-regulated HSP90 in CD90^+^HSP90^+^ cells by using mAb 11C9, to explore the downstream pathway of HSP90. After HSP90 inhibition, 314 genes up-regulated and 1271 down-regulated (Additional file 2: Fig. S2E), such as Wnt signaling (CTNNB1, CCND1, LRP5L, APC), stemness-related gene KLF4, and resistance-related gene TOP2A. By the GO and KEGG analysis, we observed that these DEGs were indeed mainly enriched in the cell cycle-, stemness- and resistance-related signaling, including p53, Hedgehog, mTOR, TGF-β, ABC transporters, and Wnt (Additional file 2: Fig. S2F & S2G). More importantly, PPI network analysis demonstrated that both HSP90AA1 and HSP90AB1 had an interaction with CTNNB1 (β-catenin) and CCND1 (Additional file 2: Fig. S2H). Thus, we next assessed the effects of HSP90 on Wnt/β-catenin signaling, since it is crucial in phenotype shaping, stemness maintenance, therapeutic resistance, etc. [20]. After HSP90 inhibition by mAb 11C9,* p*-β-catenin was significantly up-regulated, while these downstream proteins, including *p*-GSK-3β, cell cycle-related proteins (CyclinD1, C-myc), stemness-related proteins (OCT4, KLF4, Snail), were down-regulated both in MHCC97L and BEL7402 cell lines (Fig. 7A; Full-length blots/gels are shown in Additional file 3: Fig. S3). Thus, we concluded that mAb 11C9 plays anti-tumor activity by inhibiting HSP90/Wnt/β-catenin signaling in HCC (Fig. 7B).

## Discussion

The novel antibody–antigen system is necessary to circumvent tumor heterogeneity and tumor evolution in cancer therapy. Previously, we obtained a novel mAb 11C9 from the multipotent CSC mAbs library in our laboratory [14]. Here, we first verified the anti-tumor activity of mAb 11C9 in HCC. CSCs are self-renewing cells and possess stemness properties that facilitate tumor initiation, promote metastasis, and enhance therapeutic resistance [21, 22]. Therefore, the inhibition effects of mAb 11C9 on sphere formation ability, cell invasion, and cisplatin resistance were verified in two HCC cell lines, suggesting that mAb 11C9 has anti-tumor activity in HCC and may provide novel insights into the development of a therapeutic agent for HCC. However, these findings were only from in vitro experiments and remain investigated in clinical trials.

TAAs serves as the primary targets for cancer immunotherapies [23]. In the present study, in order to improve the anti-tumor activity of mAb 11C9, its candidate TAA was identified. As result, we identified the HSP90 that is recognized by mAb 11C9, previously known as an ATP-dependent molecular chaperone involved in the folding and activation of substrate proteins [24]. Additionally, HSP90 with *M*_*r*_ of ~ 95 kDa was observed to be expressed in the cell surface, being consistent with the previous reports [25]. The surface antigen expression pattern of HSP90 is also in line with the typical antigen expression pattern of CSCs [26]. As known, HSP90 is a highly conserved molecular chaperone [27], thus, it has the potential to be an ideal target for the stem cell therapy of HCC. The fixed-cell IF staining revealed that HSP90 is abundantly expressed in the membrane, cytoplasm, and nucleus, suggesting the possibility of HSP90 as an intracellular antigen. As well known, antibody-targeted therapy has traditionally targeted extracellular or secreted proteins expressed by cancer cells because the large size of most mAbs is a serious handicap for penetrating tissues and the extracellular matrix to reach their target cells [28]. Currently, intracellular antigen targeting strategies still await translation into the clinic. Here, it is because of the extensive distribution of HSP90 in HCC cell lines highlighting its great potential as an ideal therapeutic target. More remarkably, the live-cell IF staining demonstrated no obvious intracellular staining of HSP90, suggesting that these 11C9-HSP90 antibody–antigen complexes were not internalized and degraded. However, it is well known that the rapid internalization of antibody–antigen complexes protects cells from efficient antibody-mediated lysis [29], which adds to the difficulties of replicating the preclinical activity of mAb in clinical trials. Therefore, the non-internalization advantage would provide additional benefits for the 11C9-HSP90 complexes in clinical application.

Elucidating the role of TAAs in cancer progression is crucial to maximizing the value of this novel antibody–antigen system. Here, we first confirmed the predictive value of HSP90 for worse outcomes in patients with HCC, which is in agreement with previous findings [30]. A very intriguing finding here is that HSP90 was identified as a stemness-related protein in HCSCs by evaluating the co-expression of HSP90 and CSCs markers. Extensive research has also revealed that HSPs enhance the CSCs stemness [31, 32], which further supported our results. Thus, the effects of HSP90 on stemness-associated properties were explored to confirm the function of HSP90 in HCSCs. The present results were exactly as expected, that is, HSP90 promoted cell invasion, self-renewal, and tumorigenicity, as well as induced drug resistance in HCSCs. We also observed the direct inhibition effect of HSP90 on CD90^+^ or ESA^+^ cells. As CD90 and ESA are recognized markers of HCSCs [33], we concluded that HSP90 could direct affect HCSCs, being another remarkable property. These observations were completely in conformity with the classic theory of HSP90 [34]. Besides, the bioinformatics analysis further demonstrated that HSP90 mainly affects the stemness-, cell cycle-, and resistance-related genes, such as KLF4, TOP2A, and CDK1, providing additional evidence for the effects of HSP90 on stemness-associated properties.

In the present study, we also investigate the molecular mechanism of this antibody–antigen system and the potential targets in HCC. Through bioinformatics analysis, several candidate pathways came into our sights, including Hedgehog, Hippo, TGF-β, Wnt, and p53 signaling pathways. Of these, Wnt/β-catenin signaling is crucial in phenotype shaping, stemness maintenance, therapeutic resistance, etc. [20]. Thus, the effects of HSP90 on Wnt/β-catenin signaling were confirmed in HCSCs by treating with mAb 11C9. We observed that 11C9-treated cells had a lower level of *p*-GSK-3β, suggesting the inactivation of Wnt/β-catenin signaling. As the classic Wnt/β-catenin signaling [35], the suppression of GSK-3β phosphorylation, in turn, promoted the phosphorylation and ubiquitin-mediated degradation of β-catenin. The inactive Wnt/β-catenin signaling then induced the low expression of cell cycle- (CyclinD1, C-myc) and stemness-related proteins (OCT4, KLF4, Snail). In short, these results demonstrated that mAb 11C9 might play anti-tumor activity by inhibiting HSP90/Wnt/β-catenin signaling in HCC. Given that Wnt/β-catenin signaling is evolutionarily conserved and is inactive in the normal tissues, except for stem cell niches [36], targeting this signaling by the 11C9-HSP90 antibody–antigen system might provide future perspectives for the stem cell therapy of HCC.

## Conclusion

In the present study, we preliminarily proved the anti-tumor activity of mAb 11C9 in HCC cell lines. Meanwhile, HSP90 was identified as a targeted antigen of mAb 11C9. Furthermore, HSP90 was found to function as an oncogene in phenotype shaping, stemness maintenance, and therapeutic resistance by activating Wnt/β-catenin signaling in HCSCs. Our findings suggested that this novel antibody–antigen system may have the potential for tumor targeting in stem cell therapy for HCC.

### Supplementary Information

**Additional file 1.** The polymorphic short tandem repeat authentication for MHCC97L and BEL7402 cell lines.

**Additional file 2.** Supplementary method and results. **1.Supplementary method:** PKH26 staining. **2.Supplementary results: Table S1.** The correlation of HSP90 expression with clinicopathological features. **Figure S1**. Double-IF staining revealed the co-staining of HSP90 and PKH26. **Figure S2**. Bioinformatics analysis implied the potential targets of HSP90 induced the biological properties of HCSCs. CD90+HSP90+ and CD90+HSP90- cells were sorted using FACS, and then, RNA-seq was performed to detect the DEGs for the bioinformatics analysis. (A) Volcano plot illustrating genes that are differentially expressed between CD90+HSP90+ and CD90+HSP90- cells. Up-regulated genes are shown in red and down-regulated genes in green. (B) GO analysis and (C) KEGG analysis showed the representative affected pathways of DEGs. (D) Network of cell cycle/mitosis-related proteins using Cytoscape software. Next, CD90+HSP90+ cells were exposed to mAb 11C9, and then, RNA-seq was performed to detect the DEGs. (E) Volcano plot illustrating genes that are differentially expressed between CD90+HSP90+ and mAb 11C9-treated CD90+HSP90+ cells. (F) GO analysis and (G) KEGG analysis showed the representative affected pathways of DEGs. (H) Network of Wnt/β-catenin signaling-, stemness-, and cell cycle-related proteins using Cytoscape software. HSP90 genes were highlighted in yellow.

**Additional file 3.** Supplementary original image of the blotting. **Figure S1.** Full-length blots/gels of Figure 2. **Figure S2.** Full-length blots/gels of Figure 6. **Figure S3.** Full-length blots/gels of Figure 7.

## Data Availability

Sequencing results for this study were available from GEO database (GSE215984). All other dataset supporting the conclusions of this article are included within the article (and its additional file).

## Abbreviations

HCC: Hepatocellular carcinoma
mAbs: Monoclonal antibodies
TKIs: Tyrosine kinase inhibitors
CSCs: Cancer stem cells
TAAs: Tumor-associated antigens
FBS: Fetal bovine serum
Co-IP: Co-immunoprecipitation
IHC: Immunohistochemistry
FACS: Fluorescence-activated cell sorting
FITC: Fluorescein isothiocyanate
PE: Phycoerythrin
THPA: The Human Protein Atlas
DEGs: Differentially expressed genes
GO: Gene ontology
KEGG: Kyoto Encyclopedia of Genes and Genomes
PPI: Protein–protein interaction
S.E.M: Standard error of the mean
s.d.: Standard deviation
ANOVA: Analysis of variance
HCSCs: Hepatocellular cancer stem cells

## References

[1] Ferenci P, Fried M, Labrecque D, Bruix J, Sherman M, Omata M (2010). Hepatocellular carcinoma (HCC): a global perspective. J Clin Gastroenterol.

[2] Dutta R, Mahato RI (2017). Recent advances in hepatocellular carcinoma therapy. Pharmacol Ther.

[3] Sayiner M, Golabi P, Younossi ZM (2019). Disease burden of hepatocellular carcinoma: a global perspective. Dig Dis Sci.

[4] McGlynn KA, Petrick JL, El-Serag HB (2021). Epidemiology of hepatocellular carcinoma. Hepatology.

[5] Kumari R, Sahu MK, Tripathy A, Uthansingh K, Behera M (2018). Hepatocellular carcinoma treatment: hurdles, advances and prospects. Hepat Oncol..

[6] Rimassa L, Pressiani T, Merle P (2019). Systemic treatment options in hepatocellular carcinoma. Liver Cancer.

[7] Pinero F, Silva M, Iavarone M (2020). Sequencing of systemic treatment for hepatocellular carcinoma: Second line competitors. World J Gastroenterol.

[8] Chudasama V, Maruani A, Caddick S (2016). Recent advances in the construction of antibody-drug conjugates. Nat Chem.

[9] Huang A, Yang XR, Chung WY, Dennison AR, Zhou J (2020). Targeted therapy for hepatocellular carcinoma. Signal Transduct Target Ther.

[10] Yang L, Shi P, Zhao G, Xu J, Peng W, Zhang J (2020). Targeting cancer stem cell pathways for cancer therapy. Signal Transduct Target Ther.

[11] Weiner GJ (2015). Building better monoclonal antibody-based therapeutics. Nat Rev Cancer.

[12] O'Sullivan D, Henry M, Joyce H, Walsh N, Mc Auley E, Dowling P (2014). 7B7: a novel antibody directed against the Ku70/Ku80 heterodimer blocks invasion in pancreatic and lung cancer cells. Tumour Biol.

[13] O'Sullivan D, Dowling P, Joyce H, McAuley E, McCann A, Henry M (2017). A novel inhibitory anti-invasive MAb isolated using phenotypic screening highlights AnxA6 as a functionally relevant target protein in pancreatic cancer. Br J Cancer.

[14] Cao K, Pan Y, Yu L, Shu X, Yang J, Sun L (2017). Monoclonal antibodies targeting non-small cell lung cancer stem-like cells by multipotent cancer stem cell monoclonal antibody library. Int J Oncol.

[15] Hashimoto N, Tsunedomi R, Yoshimura K, Watanabe Y, Hazama S, Oka M (2014). Cancer stem-like sphere cells induced from de-differentiated hepatocellular carcinoma-derived cell lines possess the resistance to anti-cancer drugs. BMC Cancer.

[16] Shu X, Cao KY, Liu HQ, Yu L, Sun LX, Yang ZH (2021). Alpha-enolase (ENO1), identified as an antigen to monoclonal antibody 12C7, promotes the self-renewal and malignant phenotype of lung cancer stem cells by AMPK/mTOR pathway. Stem Cell Res Ther.

[17] Yang ZF, Ho DW, Ng MN, Lau CK, Yu WC, Ngai P (2008). Significance of CD90+ cancer stem cells in human liver cancer. Cancer Cell.

[18] Lin Q, Wu Z, Yue X, Yu X, Wang Z, Song X (2020). ZHX2 restricts hepatocellular carcinoma by suppressing stem cell-like traits through KDM2A-mediated H3K36 demethylation. EBioMedicine.

[19] Richichi C, Brescia P, Alberizzi V, Fornasari L, Pelicci G (2013). Marker-independent method for isolating slow-dividing cancer stem cells in human glioblastoma. Neoplasia.

[20] Xu X, Zhang M, Xu F, Jiang S (2020). Wnt signaling in breast cancer: biological mechanisms, challenges and opportunities. Mol Cancer.

[21] Lathia JD, Liu H (2017). Overview of cancer stem cells and stemness for community oncologists. Target Oncol.

[22] Chen P, Hsu WH, Han J, Xia Y, DePinho RA (2021). Cancer stemness meets immunity: from mechanism to therapy. Cell Rep.

[23] Lu L, Jiang J, Zhan M, Zhang H, Wang QT, Sun SN (2021). Targeting tumor-associated antigens in hepatocellular carcinoma for immunotherapy: past pitfalls and future strategies. Hepatology.

[24] Richter K, Buchner J (2001). Hsp90: chaperoning signal transduction. J Cell Physiol.

[25] Eustace BK, Sakurai T, Stewart JK, Yimlamai D, Unger C, Zehetmeier C (2004). Functional proteomic screens reveal an essential extracellular role for hsp90 alpha in cancer cell invasiveness. Nat Cell Biol.

[26] Yi SY, Hao YB, Nan KJ, Fan TL (2013). Cancer stem cells niche: a target for novel cancer therapeutics. Cancer Treat Rev.

[27] Sidera K, Patsavoudi E (2008). Extracellular HSP90: conquering the cell surface. Cell Cycle.

[28] Guo K, Li J, Tang JP, Tan CPB, Hong CW, Al-Aidaroos AQO (2011). Targeting intracellular oncoproteins with antibody therapy or vaccination. Sci Transl Med.

[29] Ficinska J, Van Minnebruggen G, Nauwynck HJ, Bienkowska-Szewczyk K, Favoreel HW (2005). Pseudorabies virus glycoprotein gD contains a functional endocytosis motif that acts in concert with an endocytosis motif in gB to drive internalization of antibody-antigen complexes from the surface of infected monocytes. J Virol.

[30] Xu Q, Tu J, Dou C, Zhang J, Yang L, Liu X (2017). HSP90 promotes cell glycolysis, proliferation and inhibits apoptosis by regulating PKM2 abundance via Thr-328 phosphorylation in hepatocellular carcinoma. Mol Cancer.

[31] Kabakov A, Yakimova A, Matchuk O (2020). Molecular chaperones in cancer stem cells: determinants of stemness and potential targets for antitumor therapy. Cells.

[32] Shende P, Bhandarkar S, Prabhakar B (2019). Heat shock proteins and their protective roles in stem cell biology. Stem Cell Rev Rep.

[33] Muramatsu S, Tanaka S, Mogushi K, Adikrisna R, Aihara A, Ban D (2013). Visualization of stem cell features in human hepatocellular carcinoma reveals in vivo significance of tumor-host interaction and clinical course. Hepatology.

[34] Lettini G, Lepore S, Crispo F, Sisinni L, Esposito F, Landriscina M (2020). Heat shock proteins in cancer stem cell maintenance: A potential therapeutic target?. Histol Histopathol.

[35] Chatterjee A, Paul S, Bisht B, Bhattacharya S, Sivasubramaniam S, Paul MK (2022). Advances in targeting the WNT/beta-catenin signaling pathway in cancer. Drug Discov Today.

[36] Wang W, Smits R, Hao H, He C (2019). Wnt/beta-catenin signaling in liver cancers. Cancers.

