# Supplementary material for: HSP90, as a functional target antigen of a mAb 11C9, promotes stemness and tumor progression in hepatocellular carcinoma
Source: Stem Cell Res Ther. 2023 Sep 27;14:273. doi: 10.1186/s13287-023-03453-x (PMC10523703; doi:10.1186/s13287-023-03453-x)

细胞 STR 检测报告

一、样品名称/编号：MHCC97-L

二、检测项目：STR 基因型检验

三、检测方法：用天根的基因组抽提试剂盒提取 DNA，采用 21-STR 扩增方案扩增，在 Seqstudio 型遗传分析仪上对 STR 位点和性别基因 Amelogenin 进行检测。

四、检验结果：

1.检验基本情况

多等位基因	匹配细胞系	细胞库	EV 值	匹配说明
无	MHCC97-H	ExPASy	0.95	基本匹配

多等位基因指三等位及以上基因现象。

本次检测各细胞分型结果良好。

2.样本描述

该株细胞 DNA 分型在细胞系检索中找到基本匹配细胞系，ExPASy 数据库显示细胞名为 MHCC97-H 细胞号对应 CVCL-4972，而该细胞系与 MHCC97和MHCC97-L都是同一来源的细胞，MHCC97和MHCC97-L在ExPASy 数据库中没有收录基因位点，所以匹配的是MHCC97-H即可认为被检细胞株亦与MHCC97和MHCC97-L高度同源。本次检测在该细胞系中没有发现多等位基因。

3.匹配说明

待测细胞系与收录于 ATCC，DSMZ，JCRB 和 ExPASy 数据库的细胞系 STR 数据进行比对，未收录于以上细胞库的细胞系将无法匹配。

附表：MHCC97-L 细胞的 STR 位点和 Amelogenin 位点的基因分型结果

MHCC97-L 细胞			
Marker	Allele 1	Allele 2	Allele 3
D19S433	13	14	
D5S818	12	13	
D21S11	31.2	31.2	
D18S51	13	22	
D6S1043	12	20	
AMEL	X	Y	
D3S1358	15	16	
D13S317	8	8	
D7S820	10	10	
D16S539	12	12	
CSF1PO	11	13	
Penta D	8	9	
D2S441	15	15	
vWA	14	14	
D8S1179	12	13	
TPOX	8	8	
Penta E	11	17	
TH01	9	9	
D12S391	18	18	
D2S1338	20	20	
FGA	21	24	

BNCC
BeNa Culture Collection

附图：ExPASy 数据库比对结果

Accession	Name	Nº Markers	Score	Amel	CSF1PO	D2S1338	D3S1358	D5S818	D7S820	D8S1179	D13S317	D16S539	D18S51	D19S433	D21S11	FGA	Penta D	Penta E	TH01	TPOX	vWA
NA	Query	NA	NA	x,y	11,13			12,13	10		8	12							9	8	14
CVCL_4972	MHCC97-H	8	95.24%	X,Y	11,13			12,13	10		8,11.3	12							9	8	14
CVCL_A990	chHES-74	8	72.73%	X	12,13		15,16	12,13	11,12	14,15	8	12	14,16		29,32.2	20,25	8,10	11,19	9	8	14,17
CVCL_5310	KPL-4	8	72.73%	X	11,13		16,17	11,13	9,10	11,16	12	12	15		29,32.2	19	10,14	11,12	9	8	14,19
CVCL_H722	6647	8	72.00%	X	11,13	17,25	15,17	11,13	8,10	10,13	8	10,12	13,19	14,14.2	29,30	22,24			9,9.3	8,9	14,18
CVCL_1591	NCI-H82	8	70.00%	X	11	17,24	17	12	10,13	13	8	12	14,18	13	28,30	24,25	10,12	11,12	9,9.3	11	14

分型方案及位点分布

	方案 1	方案 2	方案 3	方案 4
1	D19S433	AMEL	D2S441	TH01
2	D5S818	D3S1358	vWA	D12S391
3	D21S11	D13S317	D8S1179	D2S1338
4	D18S51	D7S820	TPOX	FGA
5	D6S1043	D16S539	Penta E	
6		CSF1PO		
7		Penta D		

检测人：张葡萄

审核人：殷世腾

签发日期：2022.10.11

河南省工业微生物菌种工程技术研究中心

Henan Engineering Research Center of Industrial Microbiology

网址：www.bncc.org.cn 电话：400-6699-833

Applied Biosystems
GeneMapper Software 6

8.24

Sample File	Sample Name	Panel	SQI	OS	SQ
C1 C1 Sample 20221011 173205.fsa	C1	MR21 v2.1			

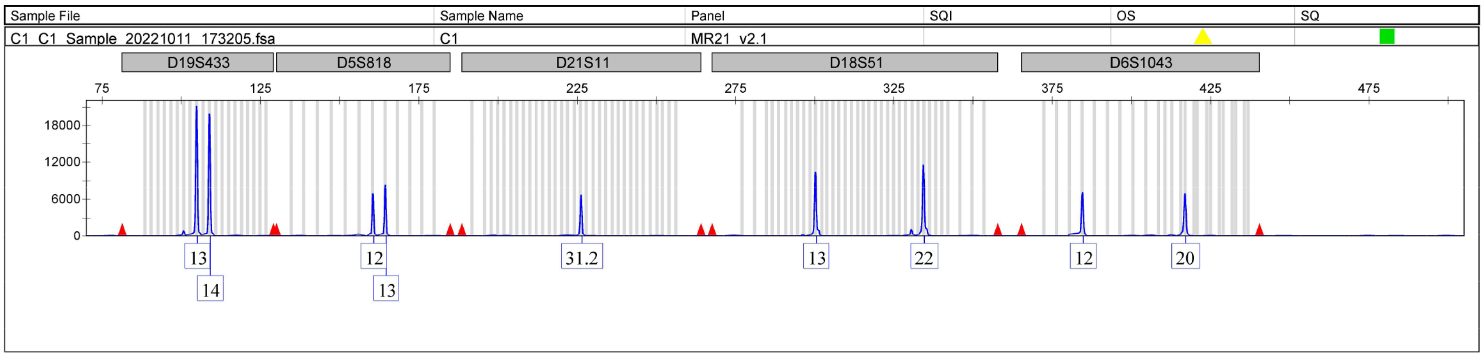

C1 C1 Sample 20221011 173205.fsa	C1	MR21 v2.1			

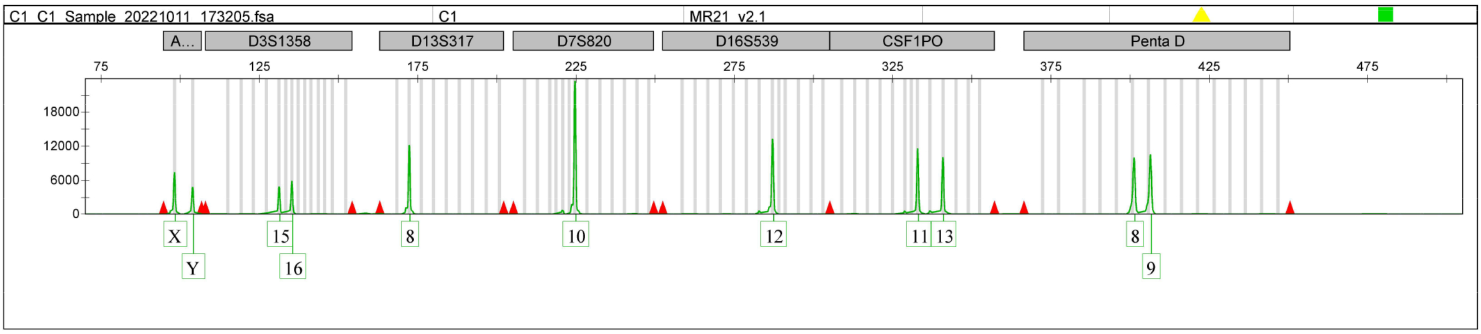

C1 C1 Sample 20221011 173205.fsa	C1	MR21 v2.1			

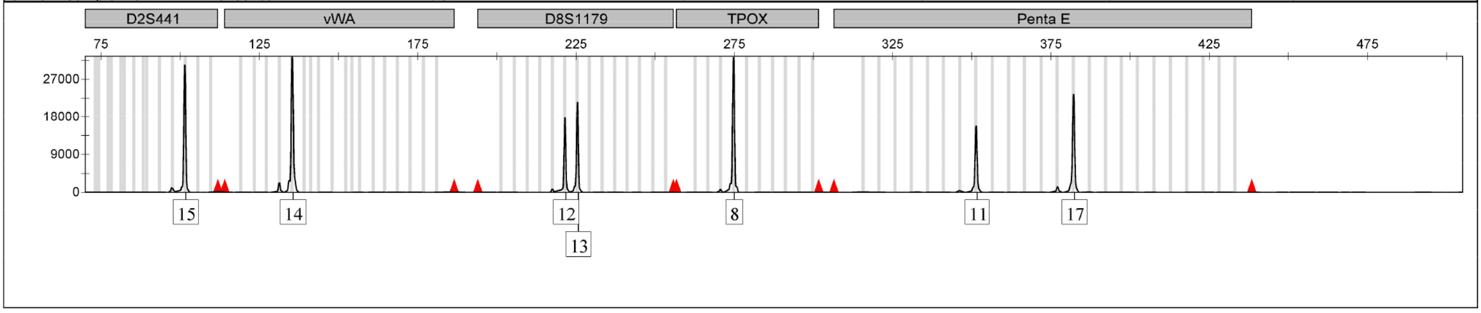

C1 C1 Sample 20221011 173205.fsa	C1	MR21 v2.1			

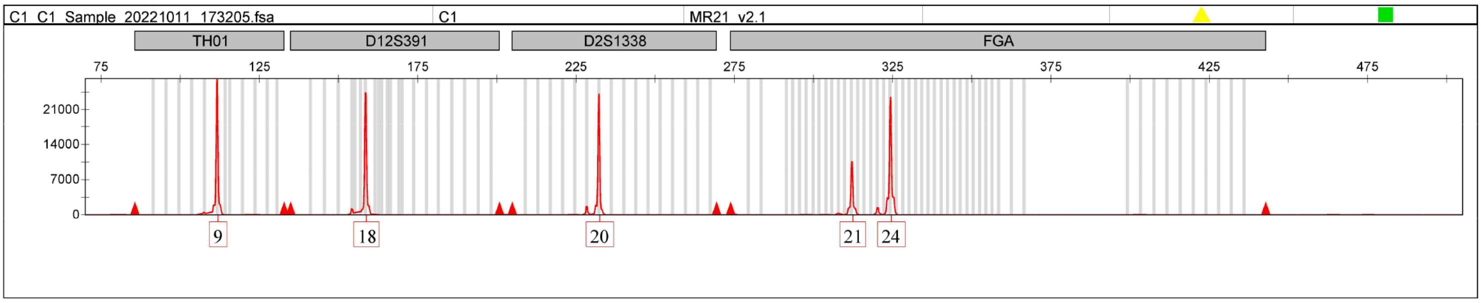

细胞 STR 检测报告

一、样品名称/编号：BEL-7402

二、检测项目：STR 基因型检验

三、检测方法：用天根的基因组抽提试剂盒提取 DNA，采用 21-STR 扩增方案扩增，在 Seqstudio 型遗传分析仪上对 STR 位点和性别基因 Amelogenin 进行检测。

四、检验结果：

1.检验基本情况

多等位基因	匹配细胞系	细胞库	EV 值	匹配说明
无	BEL-7402	ExPASy	0.80	基本匹配

多等位基因指三等位及以上基因现象。

本次检测各细胞分型结果良好。

2.样本描述

该株细胞 DNA 分型在细胞系检索中找到基本匹配的细胞系，ExPASy 数据库显示细胞名为 BEL-7402 细胞号对应 CVCL-5492。本次检测在该细胞系中没有发现多等位基因。

3.匹配说明

待测细胞系与收录于 ATCC， DSMZ， JCRB 和 ExPASy 数据库的细胞系 STR 数据进行比对，未收录于以上细胞库的细胞系将无法匹配。

BNCC BeNa Culture Collection

附表：BEL-7402 细胞的 STR 位点和 Amelogenin 位点的基因分型结果

BEL-7402 细胞			
Marker	Allele 1	Allele 2	Allele 3
D19S433	13	14	
D5S818	11	12	
D21S11	27	28	
D18S51	16	16	
D6S1043	18	18	
AMEL	X	X	
D3S1358	15	18	
D13S317	12	13.3	
D7S820	8	12	
D16S539	9	10	
CSF1PO	9	10	
Penta D	8	15	
D2S441	10	11	
VWA	16	18	
D8S1179	13	13	
TPOX	8	12	
Penta E	7	17	
TH01	7	7	
D12S391	20	25	
D2S1338	17	17	
FGA	18	21	

BNCC
BeNa Culture Collection

附图：EXPASY 数据库比对结果

CVCL_8193	EPLC-32M1	8	83.87%	X	9,10			11,12	8,12		13.3,14.3	9,10							7	8,12,13	17,18
CVCL_B3LW	HeLa-ACE2	8	83.87%	X	10			11,12	8,12,13		13.3,14.3	9,10							7	8,12	16,18,19
CVCL_B3LV	HeLa-ACE2-TMPRSS2	8	83.87%	X	10			11,12	8,12,13		13.3,14.3	9,10							7	8,12	16,18,19
CVCL_B3M4	HeLa-TMPRSS2	8	83.87%	X	10			11,12	8,12,13		13.3,14.3	9,10							7	8,12	16,18,19
CVCL_2884	D98/AH2 Clone B	8	81.48%	X	9,10			11,12	12		14	9,10							7	8,12	16
CVCL_5492	BEL-7402	8	80.00%	X	10	17	15,18	12	12	12	13.3	9,10	16	13	27,28	18,21			7	12	16,18

分型方案及位点分布

	方案 1	方案 2	方案 3	方案 4
1	D19S433	AMEL	D2S441	TH01
2	D5S818	D3S1358	VWA	D12S391
3	D21S11	D13S317	D8S1179	D2S1338
4	D18S51	D7S820	TPOX	FGA
5	D6S1043	D16S539	Penta E	
6		CSF1PO		
7		Penta D		

检测人：张葡萄

审核人：殷世腾

签发日期：2022.10.11

河南省工业微生物菌种工程技术研究中心

Henan Engineering Research Center of Industrial Microbiology

网址：www.bncc.org.cn　电话：400-6699-833

Sample File	Sample Name	Panel	SQI	OS	SQ
C3_bel-7402_Sample_20221011_175059.fsa	bel-7402	MR21_v2.1			

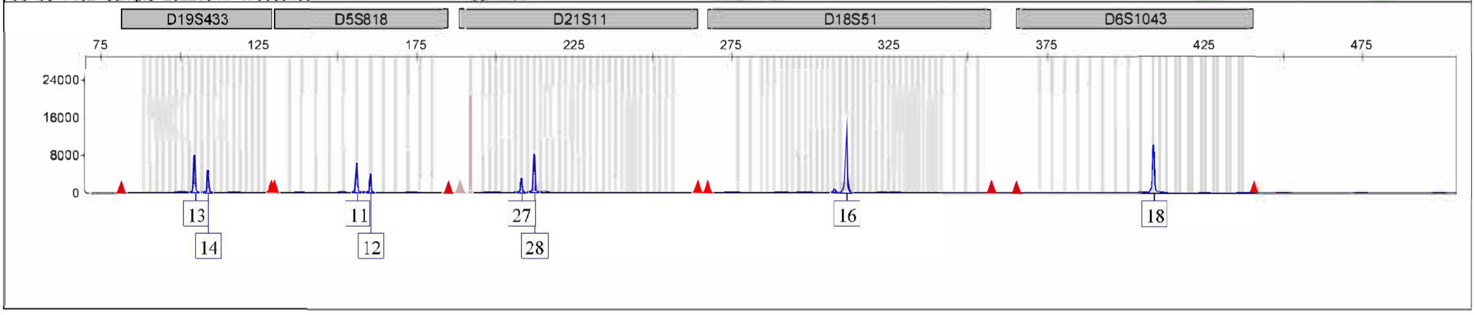

C3_bel-7402_Sample_20221011_175059.fsa bel-7402 MR21_v2.1

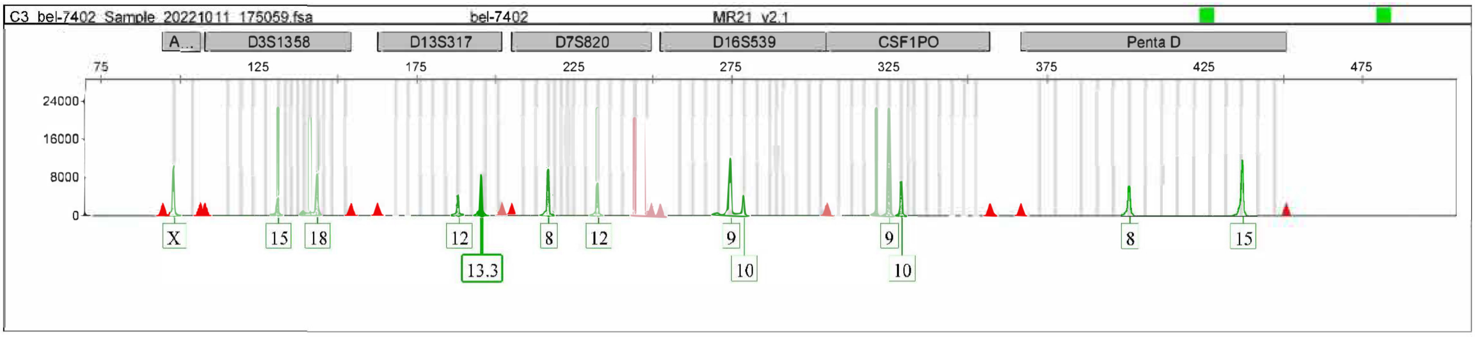

C3_bel-7402_Sample_20221011_175059.fsa bel-7402 MR21_v2.1

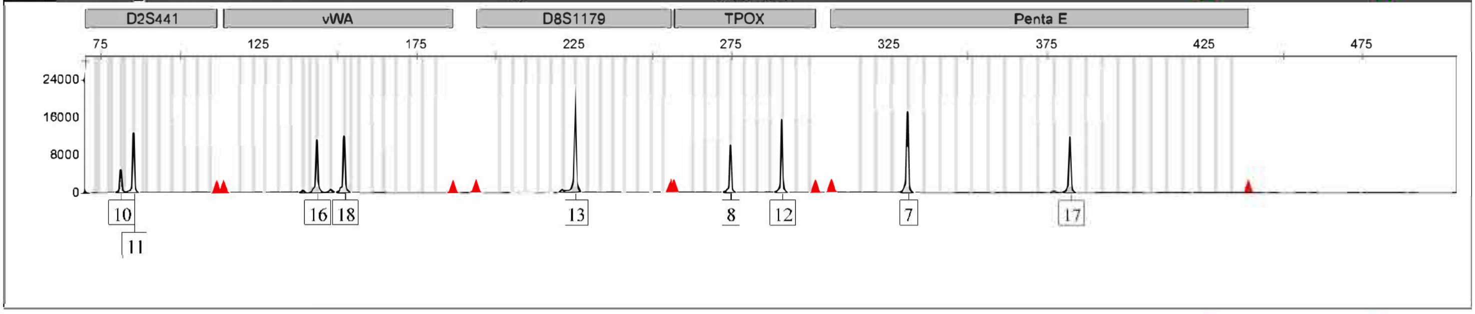

C3_bel-7402_Sample_20221011_175059.fsa bel-7402 MR21_v2.1

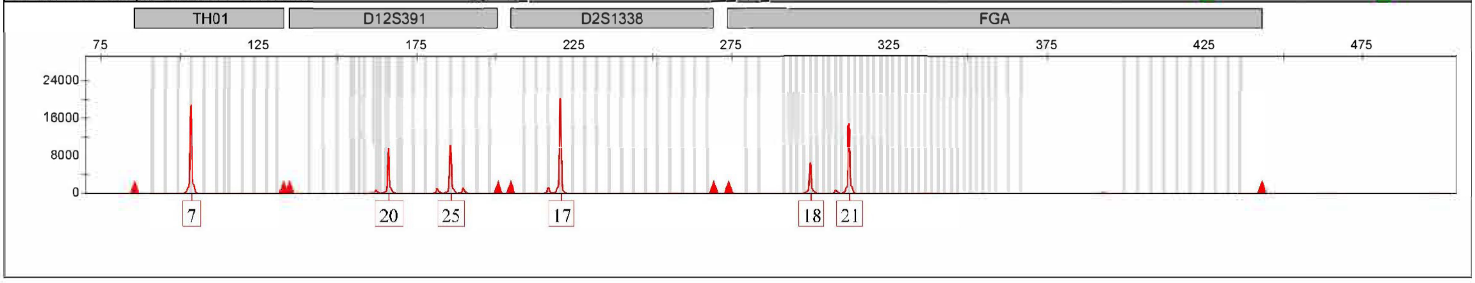

Supplement: Supplementary file 1 — Additional file 1. The polymorphic short tandem repeat authentication for MHCC97L and BEL7402 cell lines. [file 13287_2023_3453_MOESM1_ESM.pdf]
