# Supplementary material for: HSP90, as a functional target antigen of a mAb 11C9, promotes stemness and tumor progression in hepatocellular carcinoma
Source: Stem Cell Res Ther. 2023 Sep 27;14:273. doi: 10.1186/s13287-023-03453-x (PMC10523703; doi:10.1186/s13287-023-03453-x)

**Supplementary materials**

**Supplementary method**

**PKH26 staining**

Cells were dissociated and washed with serum-free DMEM/F12 medium, followed by incubation with PKH26 for 5 min. After removing excess PKH26, cells were allowed to form spheres for 7 days. Then, sphere cells were blocked with BSA, and processed for IF staining to identify co-localization of HSP90 with PKH26-labeled membranes. Commercial antibody against HSP90 as was used the primary antibody and Alexa Fluor^®^488 Goat Anti-Rabbit IgG (H+L) (green) was used as a secondary antibody.

**Supplementary results**

**Supplementary Table S1.** The correlation of HSP90 expression with clinicopathological features

| Variables |  | Case | HSP90^+^ (n=57) | HSP90^-^ (n=19) | P value |
| --- | --- | --- | --- | --- | --- |
| Gender | Male | 69 | 51 (89%) | 18 (95%) | *P*>0.05 |
|  | Female | 7 | 6 (11%) | 1 (5%) |  |
| Primary tumor (pT) | pT1 | 9 | 5 (9%) | 4 (21%) | *P*>0.05 |
|  | pT2 | 30 | 20 (35%) | 10 (53%) |  |
|  | pT3 | 36 | 31 (54%) | 5 (26%) |  |
|  | pT4 | 1 | 1 (2%) | 0 (0%) |  |
| Differentiation | High | 4 | 1 (2%) | 3 (16%) | *P*<0.05 |
|  | Moderate | 58 | 43 (75%) | 15 (79%) |  |
|  | Low | 14 | 13 (23%) | 1 (5%) |  |
| Tumor stage | Ⅰ | 8 | 6 (11%) | 2 (11%) | *P*>0.05 |
|  | Ⅱ | 30 | 19 (33%) | 11 (58%) |  |
|  | Ⅲ | 34 | 28 (49%) | 6 (32%) |  |
|  | Ⅳ | 4 | 4 (7%) | 0 (0%) |  |
| Tumor metastasis | Yes | 11 | 11 (19%) | 0 (0%) | *P*<0.05 |
|  | No | 65 | 46 (81%) | 19 (100%) |  |

**Figure S1.** Double-IF staining revealed the co-staining of HSP90 and PKH26.


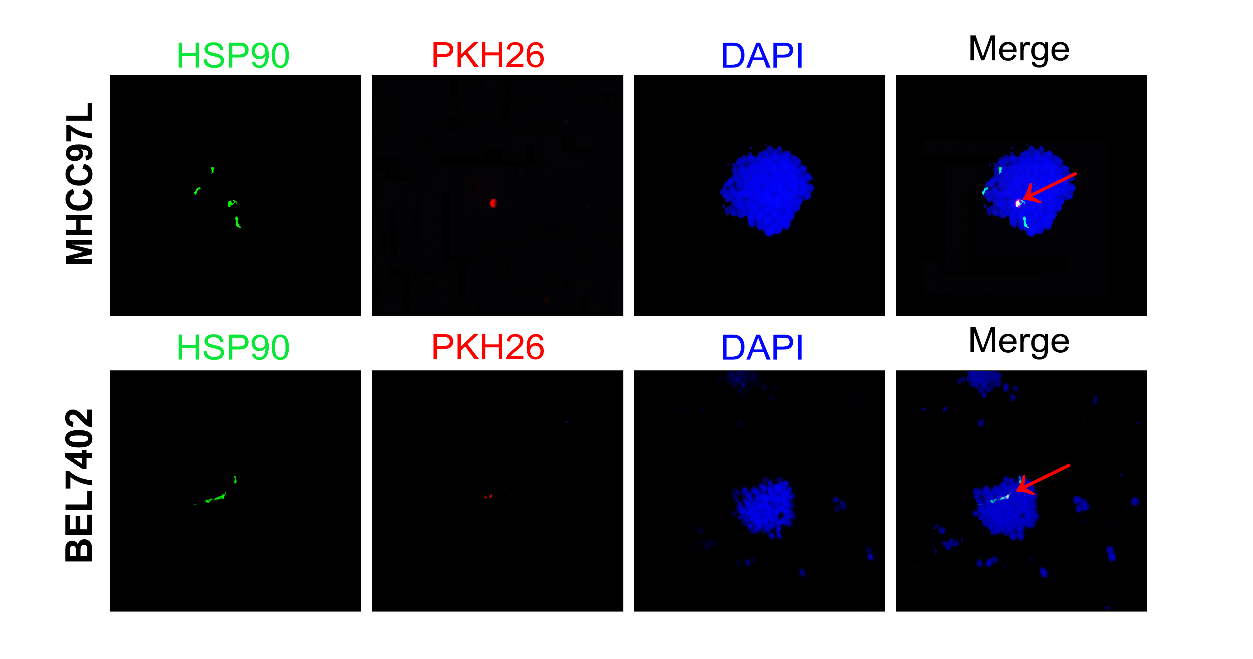


**Figure S2.** Bioinformatics analysis implied the potential targets of HSP90 induced the biological properties of HCSCs.

CD90^+^HSP90^+^ and CD90^+^HSP90^-^ cells were sorted using FACS, and then RNA-seq was performed to detect the DEGs for the bioinformatics analysis. (A) Volcano plot illustrating genes that are differentially expressed between CD90^+^HSP90^+^ and CD90^+^HSP90^-^ cells. Up-regulated genes are shown in red and down-regulated genes in green. (B) GO analysis and (C) KEGG analysis showed the representative affected pathways of DEGs. (D) Network of cell cycle/mitosis-related proteins using Cytoscape software. Next, CD90^+^HSP90^+^ cells were exposed to mAb 11C9 and then RNA-seq was performed to detect the DEGs. (E) Volcano plot illustrating genes that are differentially expressed between CD90^+^HSP90^+^ and mAb 11C9-treated CD90^+^HSP90^+^ cells. (F) GO analysis and (G) KEGG analysis showed the representative affected pathways of DEGs. (H) Network of Wnt/β-catenin signaling-, stemness-, and cell cycle-related proteins using Cytoscape software. HSP90 genes were highlighted in yellow.


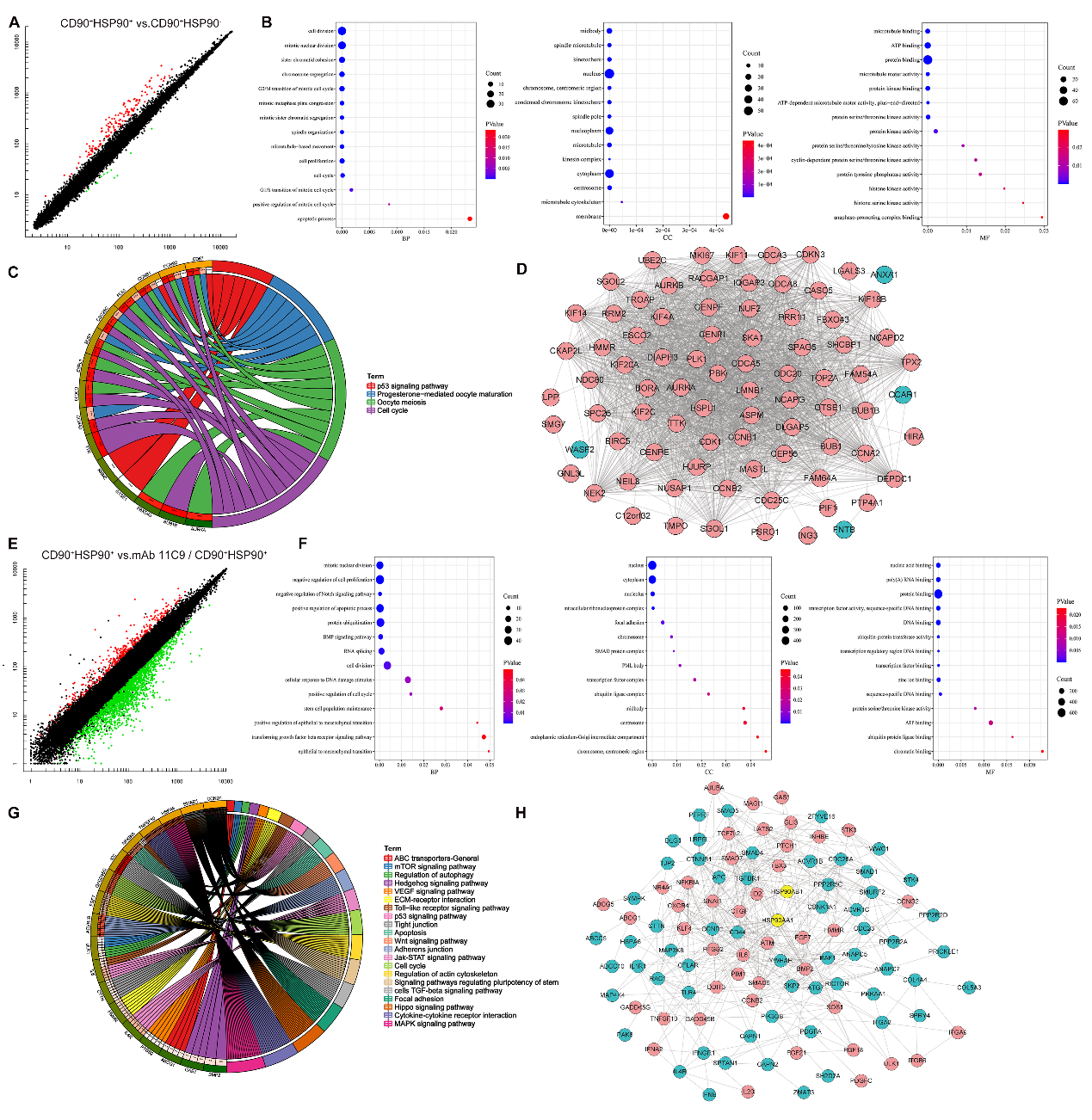

Supplement: Supplementary file 2 — Additional file 2. Supplementary method and results. 1.Supplementary method: PKH26 staining. 2.Supplementary results: Table S1. The correlation of HSP90 expression with clinicopathological features. Figure S1. Double-IF staining revealed the co-staining of HSP90 and PKH26. Figure S2. Bioinformatics analysis implied the potential targets of HSP90 induced the biological properties of HCSCs. CD90+HSP90+ and CD90+HSP90- cells were sorted using FACS, and then, RNA-seq was performed to detect the DEGs for the bioinformatics analysis. (A) Volcano plot illustrating genes that are differentially expressed between CD90+HSP90+ and CD90+HSP90- cells. Up-regulated genes are shown in red and down-regulated genes in green. (B) GO analysis and (C) KEGG analysis showed the representative affected pathways of DEGs. (D) Network of cell cycle/mitosis-related proteins using Cytoscape software. Next, CD90+HSP90+ cells were exposed to mAb 11C9, and then, RNA-seq was performed to detect the DEGs. (E) Volcano plot illustrating genes that are differentially expressed between CD90+HSP90+ and mAb 11C9-treated CD90+HSP90+ cells. (F) GO analysis and (G) KEGG analysis showed the representative affected pathways of DEGs. (H) Network of Wnt/β-catenin signaling-, stemness-, and cell cycle-related proteins using Cytoscape software. HSP90 genes were highlighted in yellow. [file 13287_2023_3453_MOESM2_ESM.docx]
