# Supplementary material for: HSP90, as a functional target antigen of a mAb 11C9, promotes stemness and tumor progression in hepatocellular carcinoma
Source: Stem Cell Res Ther. 2023 Sep 27;14:273. doi: 10.1186/s13287-023-03453-x (PMC10523703; doi:10.1186/s13287-023-03453-x)
Supplement: Supplementary file 3 — Additional file 3. Supplementary original image of the blotting. Figure S1. Full-length blots/gels of Figure 2. Figure S2. Full-length blots/gels of Figure 6. Figure S3. Full-length blots/gels of Figure 7. [file 13287_2023_3453_MOESM3_ESM.docx]

**Supplementary materials**

**Supplementary** **original image of the blotting**

**Figure S1.** Full-length blots/gels of Figure 2.

**
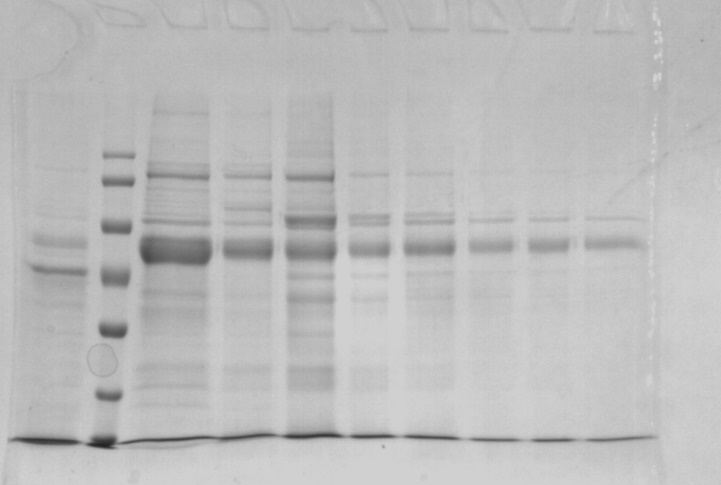
**
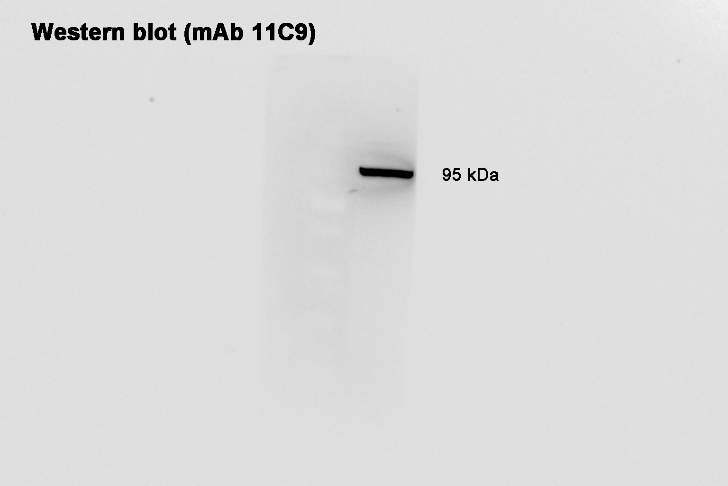


Repeat 3

Repeat 2

Repeat 1

**130 KD**

**90 KD**

**72 KD**


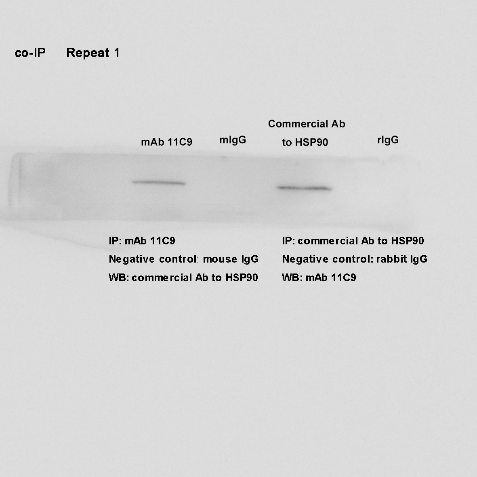

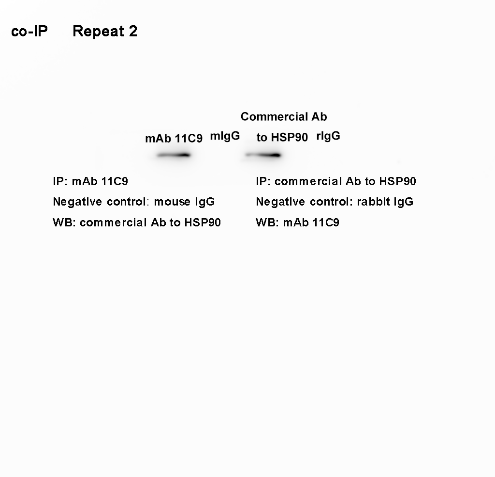

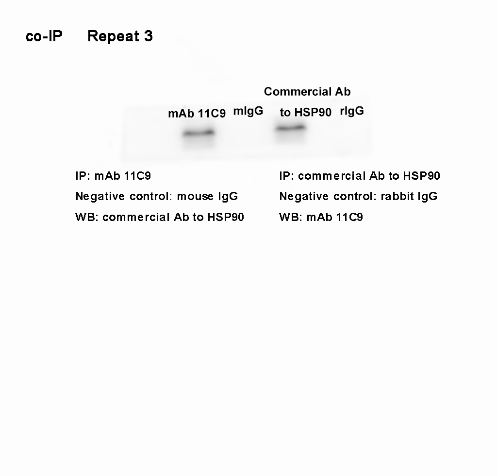


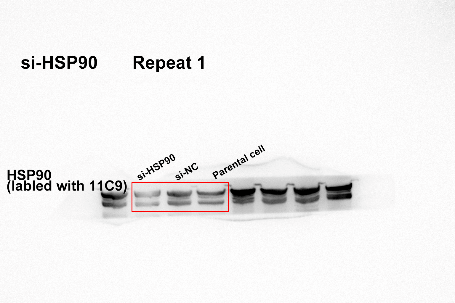

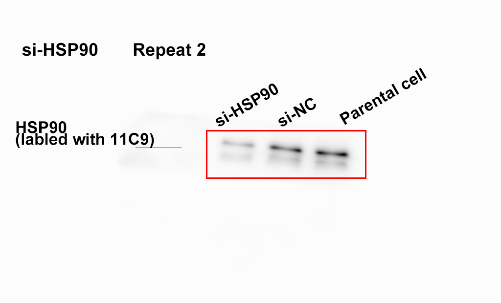

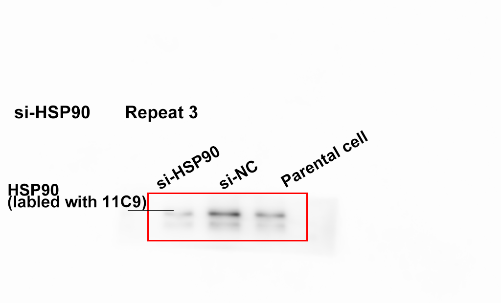


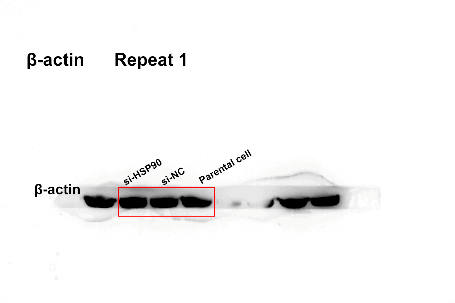
**
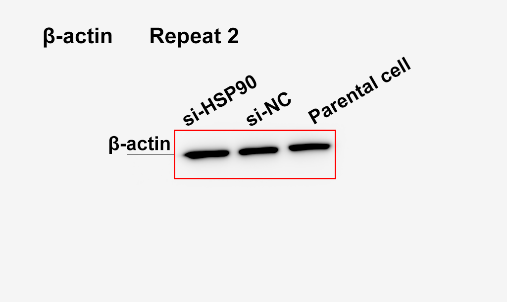

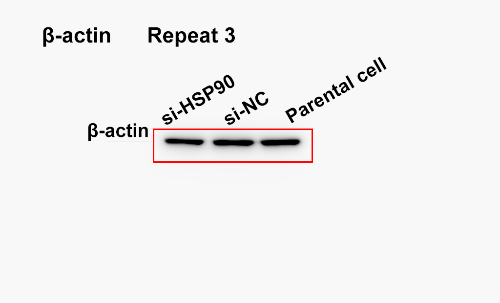
**

**Figure S2.** Full-length blots/gels of Figure 6.


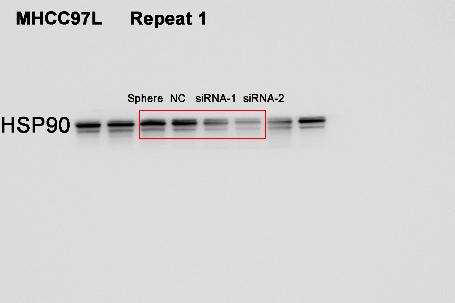

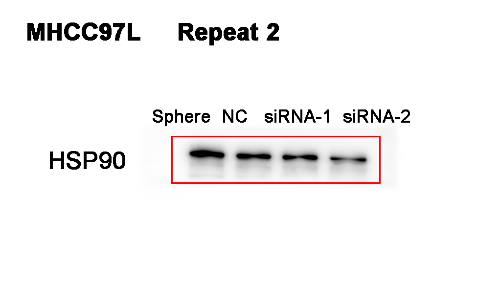

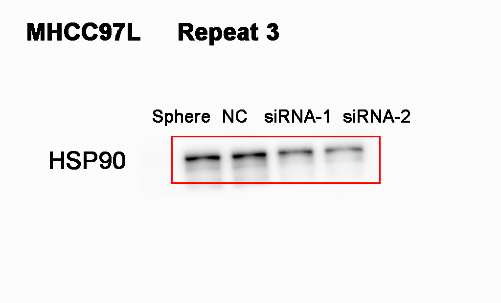


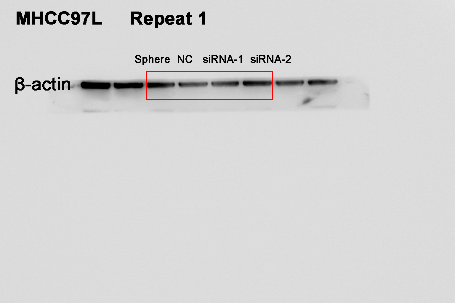
 **
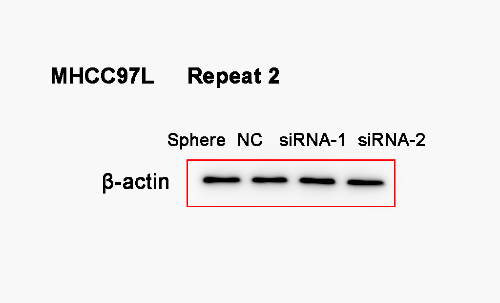

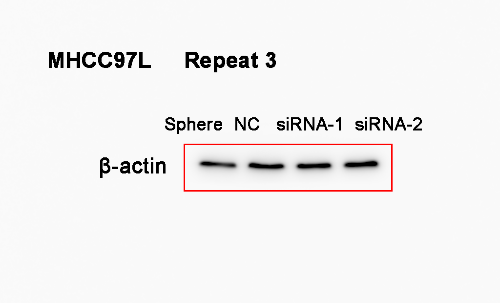
**


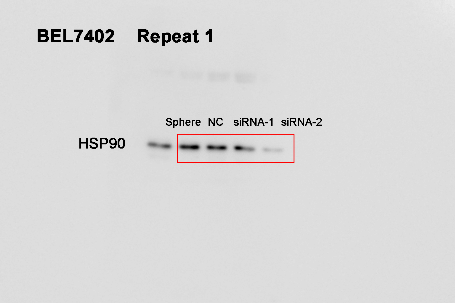

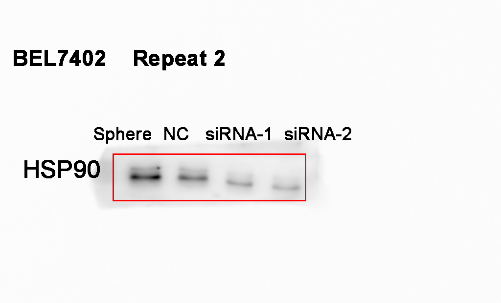

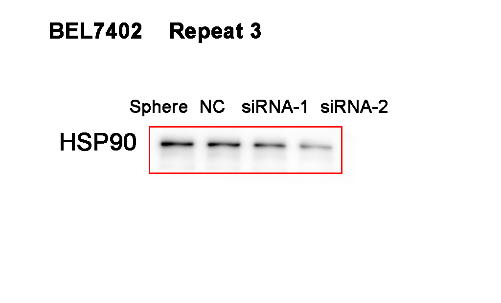


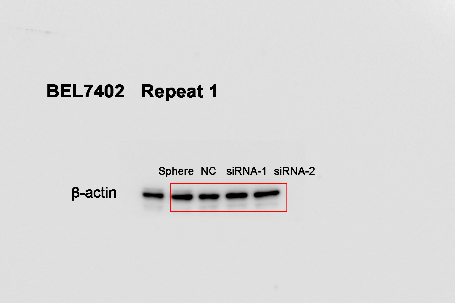

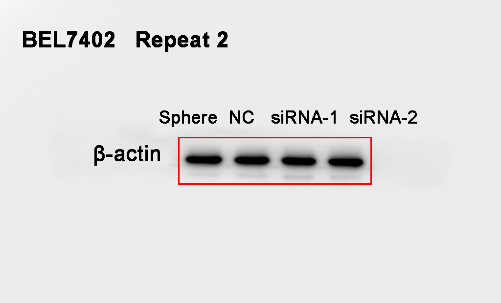

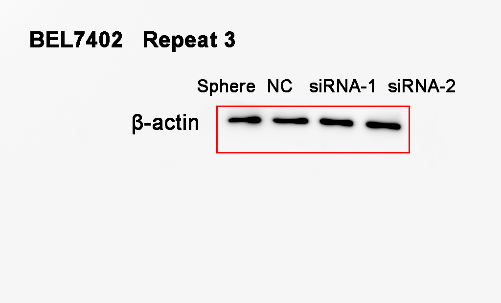


**Figure S3.** Full-length blots/gels of Figure 7.


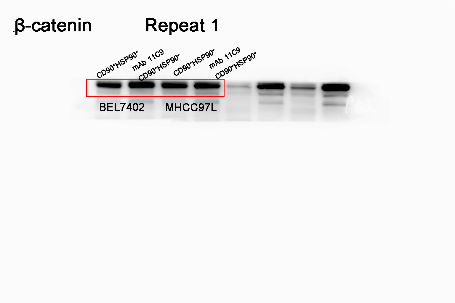

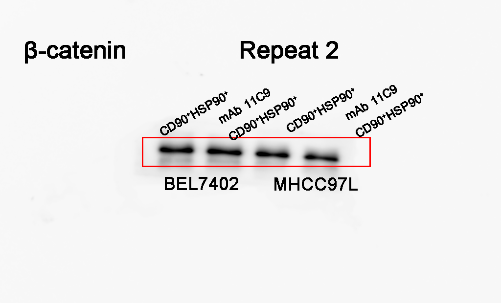

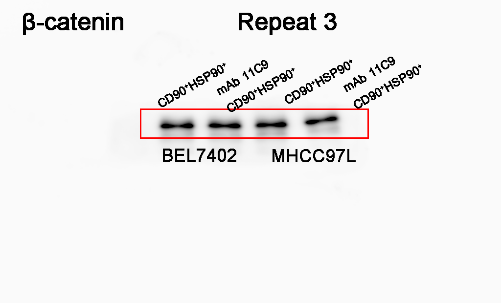


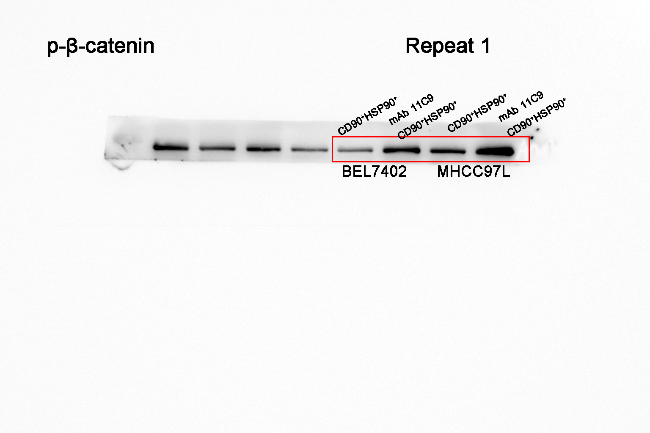

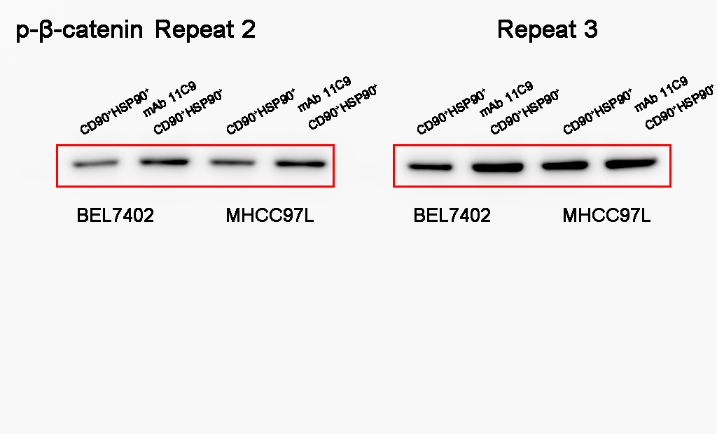


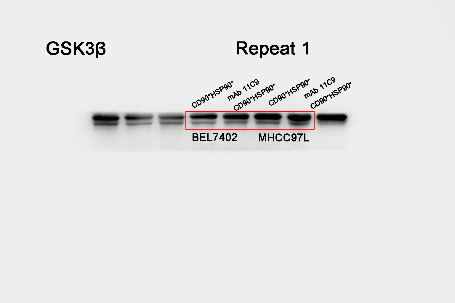

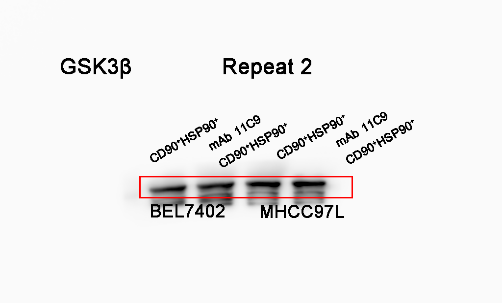

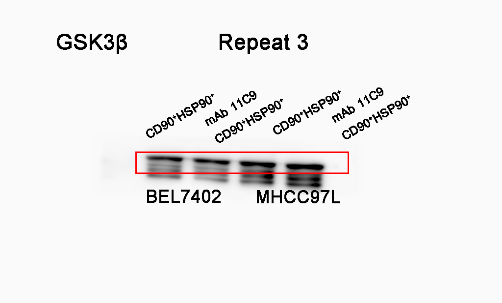


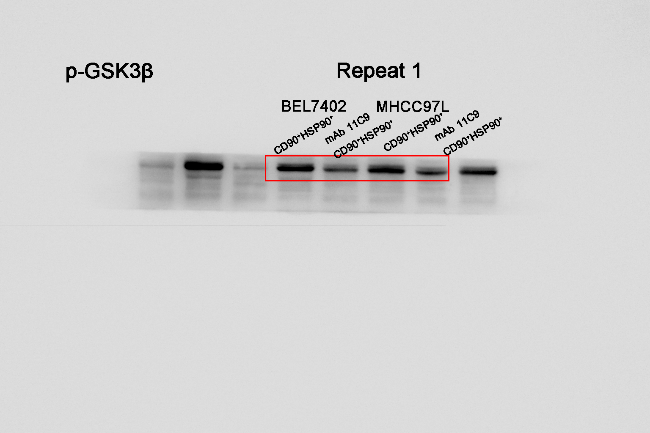

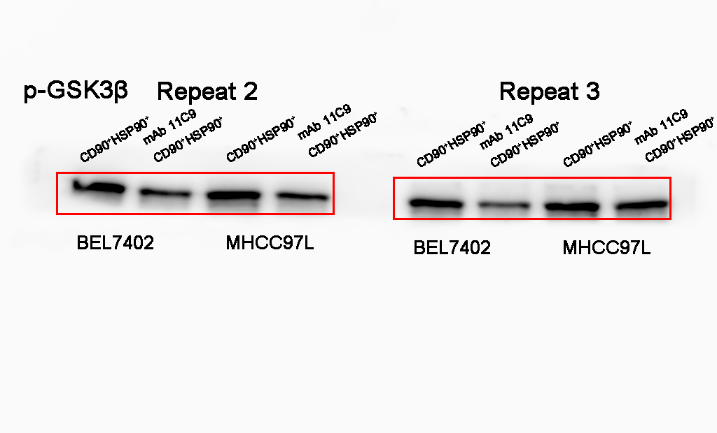


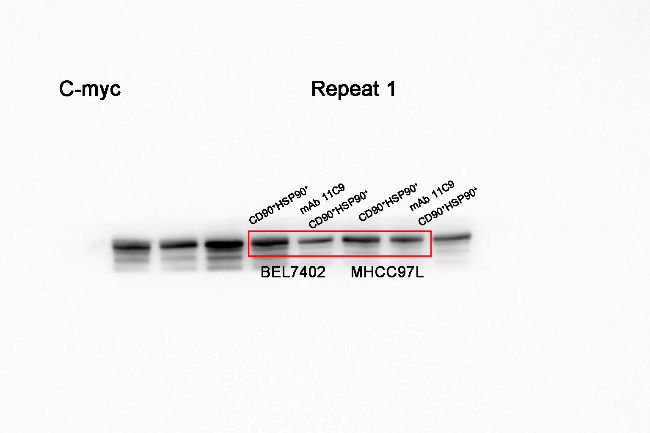

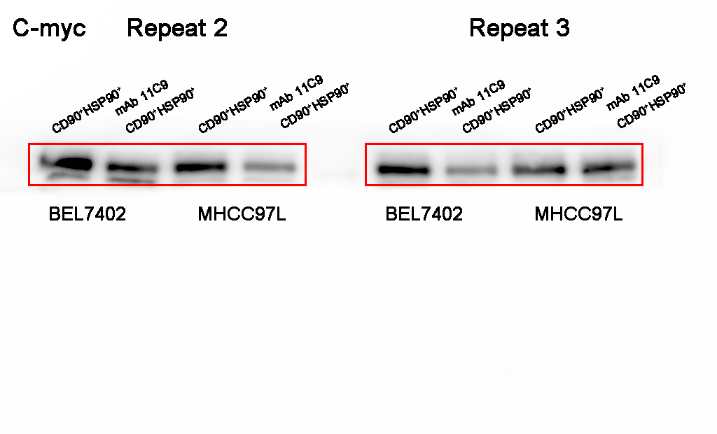


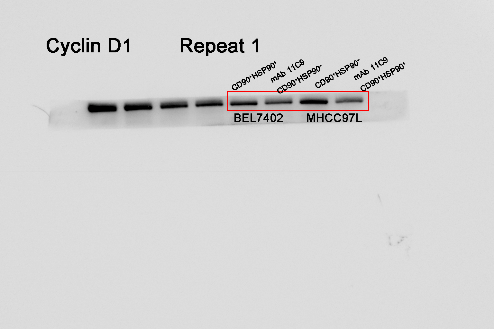

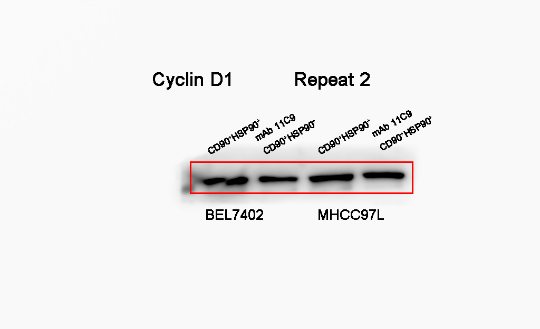

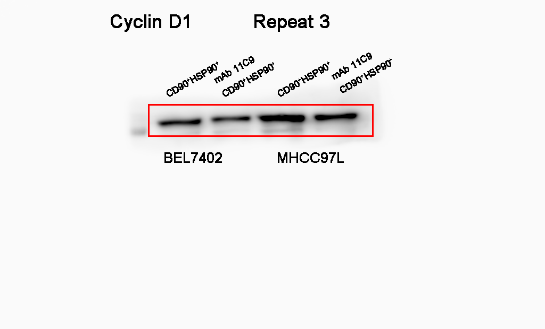


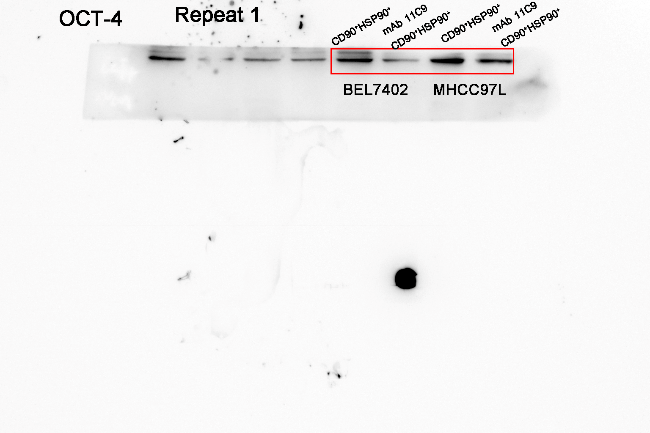

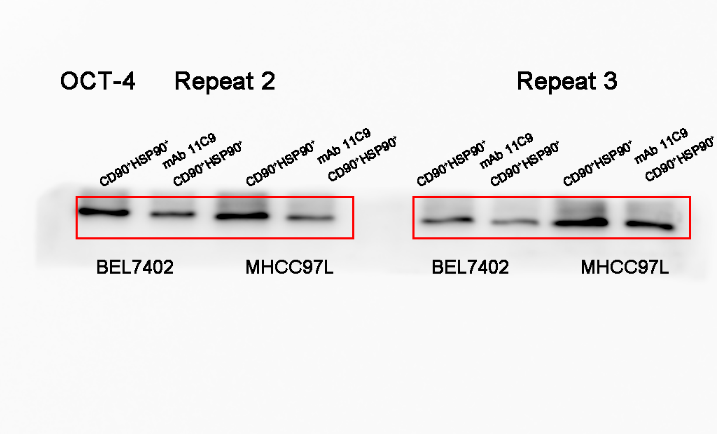


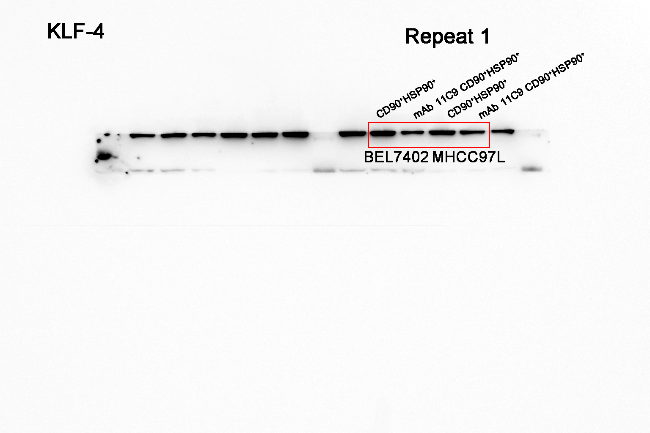

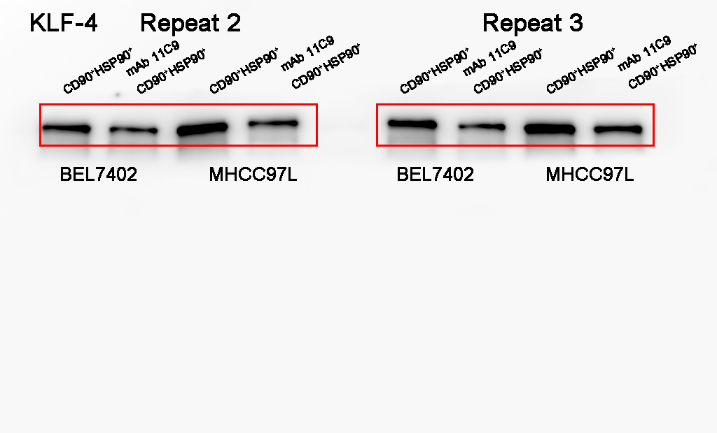


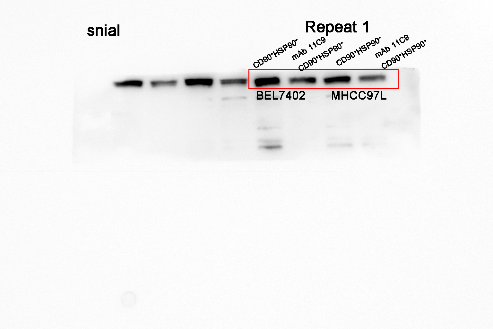

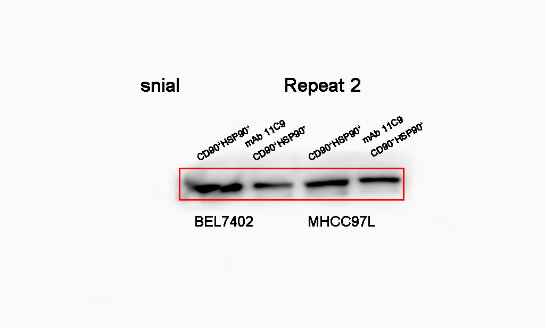

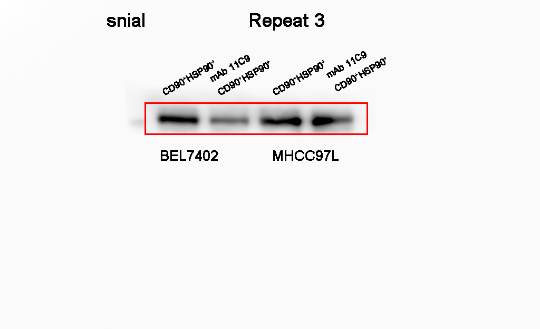


**
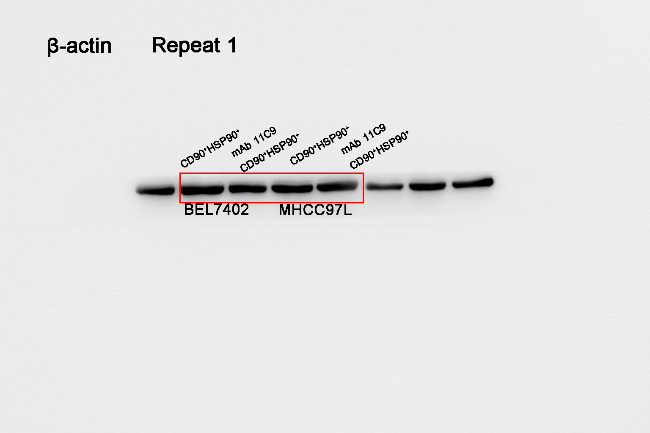

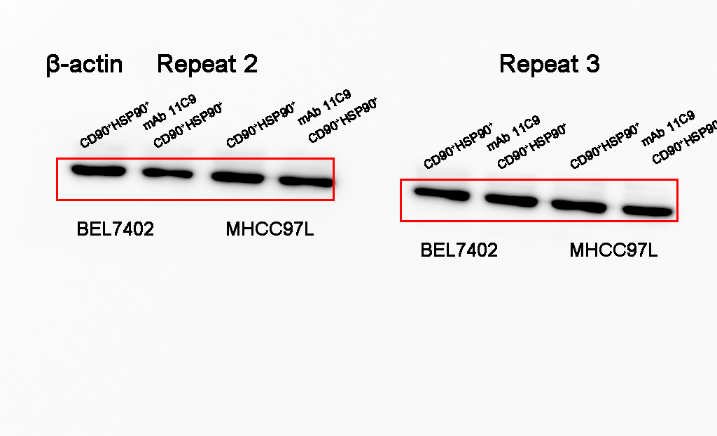
**
